# New dual functional *CYP450* gene involves in isoflavone biosynthesis in *Glycine max* L.

**DOI:** 10.1016/j.synbio.2023.01.002

**Published:** 2023-01-06

**Authors:** Yaying Xia, Chunfeng He, Su Yan, Jinyue Liu, Haijun Huang, Xue Li, Qian Su, Wenbo Jiang, Yongzhen Pang

**Affiliations:** a Key Laboratory of Plant Resources and Beijing Botanical Garden, Institute of Botany, Chinese Academy of Sciences, Beijing, 100093, China; b Institute of Animal Science, Chinese Academy of Agricultural Sciences, Beijing, 100193, China; c University of Chinese Academy of Sciences, Beijing, 100049, China

**Keywords:** Soybean, CYP82, CYP82D26, Isoflavonoids, Enzymatic activity

## Abstract

*Glycine max* L. accumulates a large amount of isoflavonoid compounds, which is beneficial for plant defense, plant-microbe symbiotic interactions, and human health. Several CYP450 subfamily genes are involved in the flavonoid biosynthetic pathway in plants. In the present study, we found 24 CYP82 subfamily genes were differentially expressed in various tissues of soybean, in *Phytophthora sojae*-infected soybean varieties and in soybean hairy roots treated with cell wall glucan elicitor. Six of them (*GmCYP82A2*, *GmCYP82A3*, *GmCYP82A4*, *GmCYP82A23*, *GmCYP82C20* and *GmCYP82D26*) were co-expressed with other known isoflavonoid pathway genes in soybean. Their enzymatic activity in yeast feeding assays showed that only GmCYP82D26 was able to convert naringenin to daidzein with both aryl migration and dehydration function. When *GmCYP82D26* was over-expressed in soybean hairy roots, the contents of the two major isoflavonoid aglycones in soybean (daidzein and genistein) were reduced, but total flavonoids were not affected. When *GmCYP82D26* was suppressed by RNAi in the hairy roots, daidzein content was decreased but genistein content was increased, with unchanged total flavonoid content. GmCYP82D26 was found to be localized in the endoplasmic reticulum at subcellular level when transiently expressed in tobacco leaf epidermis. *GmCYP82D26* gene was preferentially expressed in roots, with low expression level in other tissues in soybean. Homology modeling and molecular docking showed that GmCYP82D26 could form hydrogen bond with both HEM and naringenin at C5–OH and C4 carbonyl. All these results indicated that *GmCYP82D26* possesses new and dual enzymatic activity, which bridges the two branches (daidzein and genistein branch) of isoflavonoid pathway in soybean.

## 1 Introduction

Isoflavonoids, as plant secondary metabolites, possess a 3-phenylchroman skeleton derived from 2-phenylchroman skeleton of flavonoids, which are particularly prevalent in the Papilonoideae, subfamily of the Leguminosae [1,2]. Isoflavonoids, as signal molecules in plant-microorganism interactions, function as preformed or inducible antimicrobial or anti-insect compounds, as inducers of the nodulation genes of symbiotic *Rhizobium* bacteria, or as allelopathic agents [1,[3], [4], [5]]. In addition, isoflavonoids are structurally similar to estrogens, exerting both estrogenic and antiestrogenic properties [6,7]. Isoflavonoids from soybean have been shown to have protective effect on human health, including in immunomodulation, cognition, risk reduction of certain cancers (eg. breast and prostate cancers), cardiovascular and skin diseases, osteoporosis, ease hot flashes and obesity, as well as relief of menopausal symptoms [[7], [8], [9], [10], [11], [12], [13], [14]].

Soybean (*Glycine max* [L.] Merr.) is an important legume crop that is rich in isoflavones with diverse biological activities [15]. The three abundant isoflavones in soybean are daidzein, genistein and glycitein, and their glucosides, acetyl-glucosides and malonyl-glucosides [16]. Isoflavonoid biosynthetic pathway in soybean is derived from common flavonoid pathway (Fig. S1). Three molecules of malonyl-CoA is condensed with coumaroyl-CoA by chalcone-synthase (CHS), chalcone reductase (CHR) and chalcone isomerase (CHI) to form liquiritigenin or naringenin, respectively [[17], [18], [19]]. Liquiritigenin and naringenin are converted into the corresponding intermediates daidzein and genistein by isoflavone synthase (IFS) [20]. The pterocarpan isoflavone (glycinol) is formed from daidzein precursor by a series of subsequent reactions involving isoflavone 2′-hydroxylase (I2′H), isoflavone reductase (IFR) and pterocarpan 6α-hydroxylase (P6αH) [21,22]. Glycinol could be further prenylated by prenyltransferases [glycinol 4-dimethylallyl transferase (G4DT) to form glyceollidin I or by glycinol 2-dimethylallyl transferase (G2DT)] to form glyceollidin II, respectively [[23], [24], [25]]. Glyceollins are produced from glyceollidins by NADPH-dependent CYP450-type, cyclase(s) [26] (Fig. S1).

The cytochrome P450 (CYP450) superfamily are one of the largest gene families of enzyme proteins, which participate in various biochemical pathways, leading to the production of diverse primary and secondary metabolites [27]. Most structural genes in the flavonoid or isoflavonoid biosynthesis pathways are CYP450 family genes, CYP71D encodes flavonoid 6-hydroxylase (F6H) [28], CYP75A encodes flavonoid 3′,5′-hydroxylase (F3′5′H) [29], CYP81E encodes isoflavone 2′-hydroxylase (I2′H) and isoflavone 3′-hydroxylase (I3′H) [30,31], GmCYP93A1 encodes pterocarpan 6α-hydroxylase (P6αH) [21], CYP93B encodes flavone synthase II (FNS II) [32], and CYP93C encodes isoflavone synthase (IFS) [[33], [34], [35]]. Although total 332 full-length genes and 378 pseudogenes were identified in the soybean genome [36], function of the majority of them remain uncharacterized.

In a previous study, a genome-wide analysis of CYP450s in soybean showed that the CYP81, CYP82 and CYP93 subfamilies in soybean were grouped in one large clade [36]. Members of the CYP81, CYP82 and CYP93 subfamilies all belong to the A type CYP450 that are mainly involved in the synthesis of secondary metabolites [36], therefore, close phylogenetic relationship suggested that they may share similar function and involved in the same biosynthetic pathway. Furthermore, several genes from CYP81 and CYP93 subfamily were reported to be involved in the isoflavone biosynthetic pathway [21,[30], [31], [32], [33], [34], [35]]. For the CYP82 subfamily genes, CYP82D33 from *Ocimum basilicum* L. and CYP82D1.1 from *Scutellaria baicalensis* function as flavone 6-hydroxylase (F6H), and CYP82D2 of *S. baicalensis* as flavone 8-hydroxylase (F8H). Although twenty-four genes of the CYP82 subfamily are presented in the soybean genome, all of them are functionally unknown. In this study, we analyzed the expression profiles of all CYP82 subfamily genes from soybean, analyzed their expression patterns, isolated six candidate CYP82 subfamily genes and determined their catalytic functions in isoflavonoid pathway. We revealed that one of the six recombinant protein GmCYP82D26 functions in converting naringenin into daidzein when heterologously expressed in yeast. Our study discovered a novel and dual functional GmCYP82, which links genistein and daidzein branch in the isoflavonoid biosynthetic pathway. Our present study will enrich the diversity of the catalytic mechanism of CYP450s in soybean, as well as benefit further bioengineering of isoflavonoids in soybean or in other plant species.

## 2 Materials and methods

### 2.1 Plant materials and growth condition

*Glycine max* L. Merr. cv. Williams 82 plants were grown in growth chamber under 16 h light/8 h dark cycle at 25 °C with 65–75% relative humidity. Samples, including roots, stems and leaves of 3-week-old soybean seedlings, flowers, pods 10 days after flowering and seeds 20 days after flowering were harvested in liquid nitrogen and stored at −80 °C freezer for RNA isolation.

### 2.2 Sequence alignment of the CYP82 subfamily

Amino acid sequences of the soybean CYP82 subfamily genes and GmCYP93C5 were obtained from Phytozome 12.0 and the multiple alignment was performed using DNAMAN. Amino acid sequences of several other CYP82 subfamily genes from *Arabidopsis thaliana*, *Scutellaria baicalensis*, *Gossypium hirsutum*, *Nicotiana tabacum*, *Ocimum basilicum*, *Eschscholzia californica*, *Papaver somniferum* and *Mentha* × *piperita* were also analyzed and their protein ID were listed in Table S1. The maximum likelihood (ML) phylogeny was constructed by using the software MEGA-X with bootstrap of 1000 replicates and the LG + G + I model [37].

### 2.3 Analyses of gene expression profiling with available transcriptome/gene array data

The original expression level of soybean *CYP82* subfamily genes and six isoflavonoid pathway genes (*GmHIDH*, *GmCYP93C1v2*, *GmCYP93C5*, *GmCYP93A1*, *GmG2DT* and *GmG4DT*) in different tissues, including roots, young leaves, flowers, one cm pods, pod shells of 10 day after flowering, pod shells of 14 day after flowering, seeds of 10, 14, 21, 25, 28, 35 and 42 day after flowering, were retrieved from soybase website (https://soybase.org/soyseq/) (Table S2) and standardized by using Z-Score. Heat map was generated by using the TBtools software.

The original expression level of *GmCYP82*, *GmHIDH*, *GmCYP93C1v2*, *GmCYP93C5*, *GmCYP93A1*, *GmG2DT* and *GmG4DT* in hypocotyls infected with *Phytophthora sojae* at 72 or 120 h for 8 cultivars were retrieved from soybean affymetrix genome array (GDS3242) at https://soybase.org/expression/. These cultivars varied in resistance, including high resistance (Athow, Conrad, General and V710370), moderate resistance (PI291327 and Williams) and poor resistance (OX20-8 and Sloan). Heat map was generated by using TBtools based on the fold change of gene expression level of *P. sojae*-treated samples in compared with the mock control, with cut off value of 1 (Table S3).

The expression level of *GmCYP82*, *GmHIDH*, *GmCYP93A1*, *GmCYP93A2*, *GmCYP93C1v2*, *GmCYP93C5*, *GmG2DT* and *GmG4DT* in hairy roots of soybean W82 under the treatment of the cell wall glucan elicitor (WGE) from *P. sojae*, were retrieved from transcriptome database (GSE131686) (Table S4) [38].

### 2.4 Cloning of CYP82 subfamily genes and their expression in yeast

Roots of 3-week-old seedlings were used for total RNAs isolation by using TRIzol-A^+^ (TIANGEN, Beijing, China). The single-strand cDNAs were synthesized using Fast King gDNA Dispelling RT Super Mix (TIANGEN, Beijing, China). The sequence information of six soybean *CYP82* genes (*GmCYP82A2*, *GmCYP82A3*, *GmCYP82A4*, *GmCYP82A23*, *GmCYP82C20* and *GmCYP82D26*) were obtained based on Phytozome 12.0. The full-length open reading frames of these six genes were amplified by using the phusion high-fidelity DNA polymerase (Thermo Scientific, MA, USA) with primers listed in Table S5. For the expression of these six genes in yeast expression vector pYeDP60, their PCR products were digested with different restriction endonuclease (*Bam*H I and *Sma* I for *GmCYP82A2* and *GmCYP82C20*; *Bam*H I and *Kpn* I for *GmCYP82A3*; *Sma* I and *Kpn* I for *GmCYP82A4*; *Bam*H I and *Sac* I for *GmCYP82A23*; *Sma* I and *Sac* I for *GmCYP82D26*), and the digested fragments were then ligated to pYeDP60 digested with the same restriction endonucleases.

The six resulting expression vectors, and the empty vector pYeDP60 as control, were transformed into yeast *S. cerevisiae* WAT11. Yeast cells were grown at 28 °C for 2 days on plates with SGI + Trp medium containing 20 g/L glucose. PCR positive colonies were selected and initially grown in 1 ml SGI + Trp liquid medium with 20 g/L galactose medium at 28 °C for about 12 h. The cells were harvested by centrifugation and resuspended in the same medium to induce the expression of target proteins. Dihydromyricetin, eriodictyol, liquiritigenin, naringenin, apigenin, kaempferol, daidzein, genistein, 2′-hydroxygenistein and 3,9-dihydroxypterocarpan (ChemFaces, Wuhan, Hubei China) with final concentration of 10 μM, were then supplemented individually to the yeast cell cultures. After 24 h cultivation, the yeast cells were centrifuged, extracted twice with ethyl acetate, frozen-dried, and dissolved in 80% MeOH for further analysis.

### 2.5 Modeling and docking of six GmCYP82 proteins with naringenin

Six GmCYP82 proteins and coordinates of the HEM protoporphyrin group were modeled by using I-TASSER [39]. Naringenin from PubChem was used as input for docking by using Autodock 4.2 as described previously [40,41].

### 2.6 Subcellular localization of GmCYP82D26 in tobacco leave epidermal cells

The open reading frame of *GmCYP82D26* was cloned into plasmid pCAMBIA1302 digested with *Nco* I and *Bgl* II. The resulting GmCYP82D26-GFP fusion construct was transformed into *A. tumefaciens* strain GV3101, which was co-transformed by infiltration in *N. benthamiana*, with *A. tumefaciens* strain GV3101 containing the ER marker fused to mCherry, at a ratio of 1:1 cell culture. GFP and mCherry fluorescence were assayed after 48–72 h, using a Leica TCS SP5 confocal microscope as described previously [42]. Fluorescence for each construct was recorded separately, and the images were merged to determine co-localization.

### 2.7 *In vivo* functional characterization of *GmCYP82D26* gene in soybean hairy roots

For the functional characterization of *GmCYP82D26* gene in soybean, its open reading frame for over-expression, and the 147 bp sequence in 3′ un-translated regions for RNAi were subcloned into plasmid pTOPO-ENTR/D (Aidlab, Beijing, China). These two fragments were confirmed by sequencing and cloned respectively into plant overexpression vector pK7WG2D and RNAi vector pK7GWIWG2II 2D with Gateway LR Clonase II Enzyme Mix according to the manufacturer's protocols.

These two resulting vectors were individually transformed into *Agrobacterium rhizogenes* strain ARqua I strain, and used for infection of soybean cotyledon to generate hairy roots as described previously [38] with some modifications. The transgenic soybean hairy roots from cotyledons were transferred onto B5 medium containing 5 mg/L kanamycin, 200 mg/L cefotaxime and carbenicillin. Since these two vectors contain the GFP selection marker, thus positive hairy root lines were screened for expression of GFP under a fluorescence microscope after 2 weeks, and the hairy roots with GFP signals were collected after 14 days cultivation and ground in liquid nitrogen for RT-qPCR analysis. These hairy root samples were also extracted with 80% methanol with sonication for 1 h, and the extracts were filtered using 0.22 μm columns before UPLC analysis.

### 2.8 Determination of gene expression level by RT-qPCR

Gene expression levels in various tissues, including roots, stems, leaves, flowers, pods 10 days after flowering and seeds 20 days after flowering, or hairy roots, were determined by RT-qPCRs. Total RNAs from these tissues were isolated and reverse transcribed into cDNA as above. All RT-qPCRs were carried out using a 2 × RealStar Green Fast Mixture (GeneStar, Shanghai, China) on ABI 7500 real-time Detection System (Applied Biosystems, Foster City, CA, USA). Gene-specific primers were shown in Table S5. The PCRs were carried out as follows: 94 °C for 30 s, followed by 40 cycles of 94 °C for 5 s, and 60 °C for 34 s. The transcript levels of each gene were calculated by relative quantification using the 2^−ΔΔCT^ method and normalized with *SUB3* gene as a reference [43]. Data were obtained from biological triplicates with technical triplicates.

### 2.9 Detection of flavonoid compounds by using UPLC-MS/MS

Enzymatic reactions and hairy root extracts were filtered and analyzed on UPLC (Agilent 1290 Infinity II, Santa Clara, CA, United States) with a Eclipse XDB-C18 column (4.6 × 150 mm, 5-μm) maintained at 30 °C. Separation was performed with 0.1% (v/v) formic acid/water (A) and 100% methanol (B) at 1 mL/min flow rate. The linear gradient was 5%–80% B over 16 min, 80%–100% B from 16 to 18 min, 100% B for 2 min, and equilibrated at 5% B for 1 min. The flavonoid compounds were detected at 280 nm with a diode array detector. Flavonoids contents were measured by comparing area of individual peak with standard curves obtained with standard compounds (daidzein and genistein).

The enzymatic products were also detected on LC-MS/MS on an ion-trap TOF mass spectrometer attached to UPLC (Agilent, Santa Clara, CA, United States). Separation was performed on a Eclipse Plus C18 column (2.1 mm × 100 mm, 1.8-μm) using the same solvent system as described above for UPLC. The linear gradient was 5%–60% B over 5 min, 60%–100% B from 5 to 10 min, 100% B for 2 min, and equilibrated at 5% B for 0.1 min. The flow rate was maintained at 0.4 mL/min, and the column temperature was maintained at 30 °C. Flavonoid compounds were detected under negative mode, and the *m/z* spectra were collected from 500 to 1000 as described previously [44].

### 2.10 Measurement of flavonoid content in soybean hairy roots

Total 50 μL flavonoid extracts extracted with 80% methanol as above was sequentially mixed with 200 μL ddH_2_O and 15 μL 5% NaNO_2_, and stood for 5 min. Then 15 μL 10% AlCl_3_ was added, mixed well and stood for 10 min. Afterwards, 100 μL 1 M NaOH and 120 μL ddH_2_O were added, mixed well and stood for 15 min. Finally, 180 μL reaction was measured at wavelength of 510 nm using the microplate reader spectrophotometrically at 510 nm. Absorbance values were converted into total flavonoids using a standard curve with quercetin.

## 3 Results

### 3.1 Sequence analyses of soybean CYP82 subfamily genes

CYP82 subfamily are a large group among CYP450 families, total 24 soybean CYP82 subfamily genes were identified in the soybean genome, including 11 genes of the GmCYP82A subfamily (*GmCYP82A2*, *GmCYP82A3*, *GmCYP82A4*, *GmCYP82A18*, *GmCYP82A19*, *GmCYP82A20*, *GmCYP82A22*, *GmCYP82A23*, *GmCYP82A24*, *GmCYP82A25* and *GmCYP82A26*), four of GmCYP82C subfamily (*GmCYP82C1*, *GmCYP82C18*, *GmCYP82C20*, and *GmCYP82C21*), five of GmCYP82D subfamily (*GmCYP82D25*, *GmCYP82D26*, *GmCYP82D27*, *GmCYP82D28* and *GmCYP82D29*), one of GmCYP82J subfamily (*GmCYP82J3*) and three of GmCYP82L subfamily (*GmCYP82L4*, *GmCYP82L5* and *GmCYP82L6*). Multiple sequence alignment at amino acid level showed that all 24 members shared high sequence similarity in two characteristic regions, E-R-R triade and the heme-binding domain (FXXGXRXCXG) (Fig. S2). The E-R-R triade containing K-helix (KETLR) and PERF conserved sequence, was reported to be involved in locking the heme pocket into position to ensure stable interaction of heme with CYP450 protein [27,45].

A phylogenetic tree with all CYP82 protein sequences from nine plant species (*G. max*, *Arabidopsis thaliana*, *Scutellaria baicalensis*, *Gossypium hirsutum*, *N. tabacum*, *Ocimum basilicum*, *Eschscholzia californica*, *Papaver somniferum* and *Mentha × piperita*) showed that these CYP82 proteins could be clustered into seven clades (B–H) and GmCYP93C5 was clustered into a separate clade A (Fig. S3). Among them, all 11 members of the soybean CYP82A subfamily from soybean were clustered in clade E (Fig. S3). The soybean CYP82D subfamily were separated into three clades (F–H), with GmCYP82D26 and GmCYP82D27 formed another small clade G, and GmCYP82D29 in clade H. All members of the soybean CYP82C subfamily clustered together into clade H (Fig. S3). GmCYP82J3 and three members of GmCYP82L subfamily (GmCYP82L4, GmCYP82L5 and GmCYP82L6), NtCYP82E4v1 from *N. tabacum* and AtCYP82G1 from *Arabidopsis* were clustered together into cluster B (Fig. S3). PsCYP82N3, PsCYP82N4 and PsCYP82Y1 from *P. somniferum*, and EcCYP82N2v2 from *E. californica* were clustered together into clade D. In addition, EcCYP82B1 from *E. californica* was clustered into a single cluster C (Fig. S3). It is obvious that members of the same subfamily and the same plant species tend to cluster together in the phylogenetic tree.

### 3.2 *CYP82* subfamily genes were differentially expressed in soybean

**Fig. 1:**
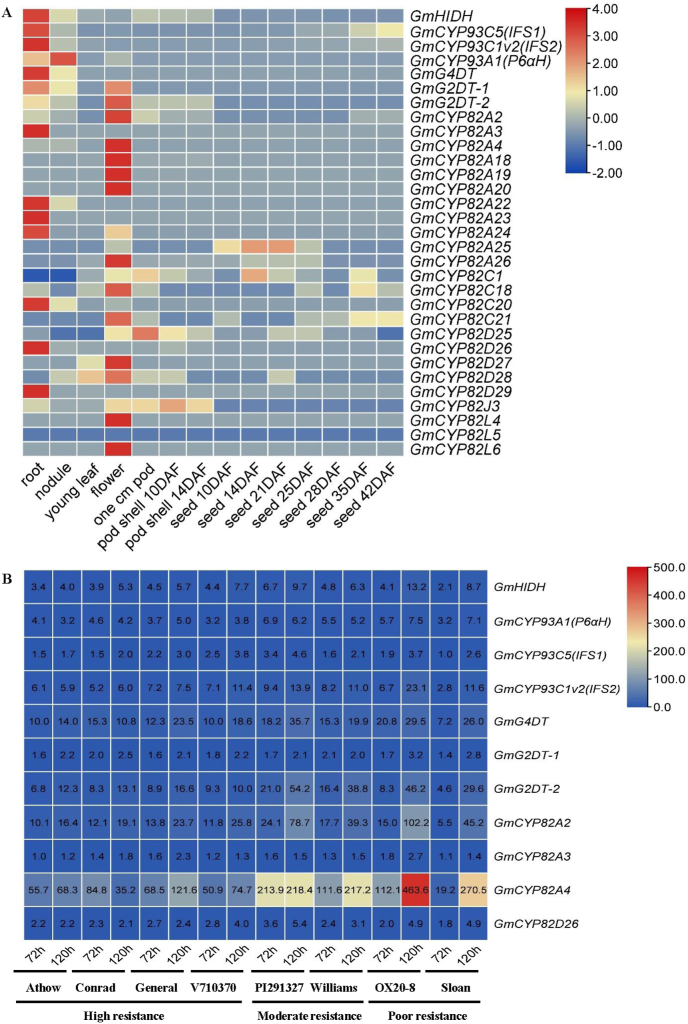
Expression profiles of soybean CYP82 subfamily genes and isoflavone pathway genes in various tissues and under *P. sojae* treatment. (A) Expression profiles of soybean CYP82 subfamily genes and isoflavone pathway genes in vaious tissues of soybean. RNA-Seq data were retrieved from soybase website and standardized using Z-Score. Heat map was generated using TBtools, and the color scale indicates gene expression level (blue indicates the expression level is low; red indicates the expression level is high). (B) Expression levels of CYP82 subfamily genes and isoflavone pathway genes that were induced by *P. sojae* treatment in 8 soybean cultivars with different resistance against *P. sojae*. The data were retrieved from soybean affymetrix genome array (GDS3242). Heat map was generated based on the fold change of gene expression levels treated with *P. soja*e compared with mock control, and the number indicates fold change (blue indicates the fold change is low and red indicates the fold change is high).

The expression profiles of soybean *CYP82* subfamily genes in soybean were analyzed based on the transcriptome data retrieved from soybase (https://soybase.org/soyseq/). Total twelve genes (*GmCYP82A2*, *GmCYP82A4*, *GmCYP82A18*, *GmCYP82A19*, *GmCYP82A20*, *GmCYP82A26*, *GmCYP82C18*, *GmCYP82C21*, *GmCYP82D27*, *GmCYP82D28*, *GmCYP82L4* and *GmCYP82L6*) were relatively highly expressed in flowers, seven genes (*GmCYP82A3*, *GmCYP82A22*, *GmCYP82A23*, *GmCYP82A24*, *GmCYP82C20*, *GmCYP82D26*, and *GmCYP82D29*) were relatively highly expressed in roots. In addition, *GmCYP82A25* was highly expressed in 1 cm pods and in seeds of 14 and 21 days after flowering, *GmCYP82C1* in seeds of 14 days after flowering, *GmCYP82D25* and *GmCYP93A41* in seeds of 14 days after flowering, *GmCYP82J3* in pod shell of 10 days after flowering, whereas *GmCYP82L5* was not expressed in all tested tissues (Fig. 1A and Table S2). These data suggested that CYP82 subfamily genes function in tissue-specific manner in soybean.

Genes in the same pathway are coordinately regulated at transcriptional level, and CYP450s have been shown to be co-expressed and regulated with other known genes in the same pathway in *Arabidopsis* and soybean [35,46]. In this study, we also performed co-expression analysis with soybean *CYP82* subfamily genes and known isoflavonoid pathway genes based on soybean affymetrix genome array (GDS3242) (Table S3) and transcriptome data (GSE131686) (Table S4). In a previous study, it was reported that the final products of the isoflavonoid biosynthesis in soybean, glyceollin I, II, and III were accumulated in hairy roots of soybean W82 under the treatment of the cell wall glucan elicitor (WGE) from *P. sojae*, whereas no glyceollins were detected from the mock control [38]. It was found that the five isoflavonoid pathway genes *GmHIDH*, *GmCYP93A1*, *GmCYP93C1v2*, *GmCYP93C5* and *GmG4DT* were up-regulated at different level under WGE treatment (Table S4), therefore, it was likely that uncharacterized CYP82 subfamily genes that were up-regulated under WGE treatment might be involved in isoflavonoid pathway, and these soybean CYP82 subfamily genes, namely *GmCYP82A2*, *GmCYP82A3*, *GmCYP82A4*, *GmCYP82A23* and *GmCYP82C20* were activated by WGE (Table S4)*.*

We also performed expression analysis of soybean *CYP82* subfamily genes and known isoflavonoid pathway genes under infection of *P. sojae* in soybean cultivars varying in *P. sojae* resistance, based on the data retrieved from soybean affymetrix genome array (GDS3242) (Fig. 1B and Table S3). It was revealed that *GmHIDH*, *GmCYP93A1*, *GmCYP93C1v2*, *GmCYP93C5*, *GmG2DT* and *GmG4DT* were up-regulated after *P. sojae* treatment, and four *GmCYP82* genes (*GmCYP82A2*, *GmCYP82A3*, *GmCYP82A4* and *GmCYP82D26*) were up-regulated after *P. sojae* treatment among 8 soybean cultivars (Fig. 1B and Table S3). In addition, *GmCYP82A2*, *GmCYP82A3*, *GmCYP82A4*, *GmCYP82A23*, *GmCYP82C20* and *GmCYP82D26* were preferentially expressed in roots or in flowers, which is similar with other five isoflavonoid pathway genes (*GmHIDH*, *GmCYP93C1v2*, *GmCYP93C5*, *GmCYP93A2* and *GmG4DT*) (Fig. 1A and Table S2). Combined with the above expression profiles of the soybean CYP82 subfamily genes, we found that six *CYP82* genes (*GmCYP82A2*, *GmCYP82A3*, *GmCYP82A4*, *GmCYP82A23*, *GmCYP82C20* and *GmCYP82D26*) were co-expressed with isoflavonoid pathway genes and they were induced by WGE and *P. sojae* treatment, thus these six genes were selected as candidates for further analysis*.*

### 3.3 Catalytic function of six candidate GmCYP82 proteins

Six candidate GmCYP82 proteins (GmCYP82A2, GmCYP82A3, GmCYP82A4, GmCYP82A23, GmCYP82C20 and GmCYP82D26) were expressed in yeast strain WAT11, which contains an *Arabidopsis* NADPH cytochrome P450 reductase gene (*ATR1*) [47]. The yeast strains expressing each individual protein were fed with 10 different type of representative flavonoid compounds as substrate, including flavanones (dihydromyricetin, eriodictyol, liquiritigenin and naringenin), flavone (apigenin), flavonol (kaempferol), isoflavones (daidzein, genistein and 2′-hydroxygenistein, and 3,9-dihydroxypterocarpan) (Table S6). The yeast cells containing the pYeDP60 vector was used as control with each substrate. The feeding assays were performed for 24 h, and the cell cultures were harvested, extracted with ethyl acetate, and analyzed on UPLC.

**Fig. 2:**
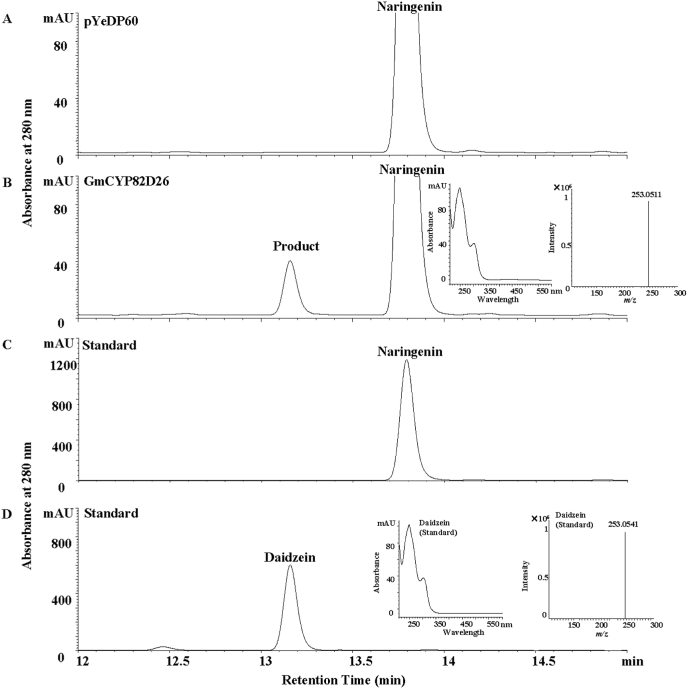
Detection of enzymatic product of the recombinant GmCYP82D26 protein with naringenin as substrate by UPLC. (A) UPLC profiles of the enzymatic product with the empty vector pYeDP60. (B) UPLC profile, UV spectrum and mass spectrum of the enzymatic product of the recombinant GmCYP82D26 protein feed with naringenin in yeast cell culture. (C) UPLC profiles of naringenin standard. (D) UPLC profile, UV spectrum and mass spectrum profile of daidzein standard.

As a result, one new product was detected at retention time of 13.1 min in the feeding assay expressing GmCYP82D26 protein with naringenin as substrate (Fig. 2 B), whereas no new product was found in the assay for the control (Fig. 2A). The retention time, UV spectra, molecular ion (M − H) (253.0511) and tandem MS/MS spectrum of the enzymatic product of GmCYP82D26 (Fig. 2B, Fig. S4A) were identical to the authentic daidzein standard (Fig. 2D and Fig. S4B), therefore, this product was identified to be daidzein. Furthermore, the product daidzein was also confirmed when microsomal protein was used in the enzymatic assay with naringenin as substrate (Fig. S5).

However, no new peak was produced for the other five proteins (GmCYP82A2, GmCYP82A3, GmCYP82A4, GmCYP82A23 and GmCYP82C20) with all the tested substrates (Table S6, Fig. S6-1 to S6-10). Although we once detected a small peak for GmCYP82A3 with liquiritigenin as substrate, the result was not repeatable in the follow-up experiment, GmCYP82A3 was thus regarded as inactive towards liquiritigenin (Table S6).

In summary, GmCYP82D26 showed activity towards naringenin to produce daidzein with strict substrate selection, while other five soybean CYP82 subfamily members (GmCYP82A2, GmCYP82A3, GmCYP82A4, GmCYP82A23 and GmCYP82C20) showed no activity toward any of these tested ten substrates.

### 3.4 Over-expression and suppression of *GmCYP82D26* in soybean hairy roots

**Fig. 3:**
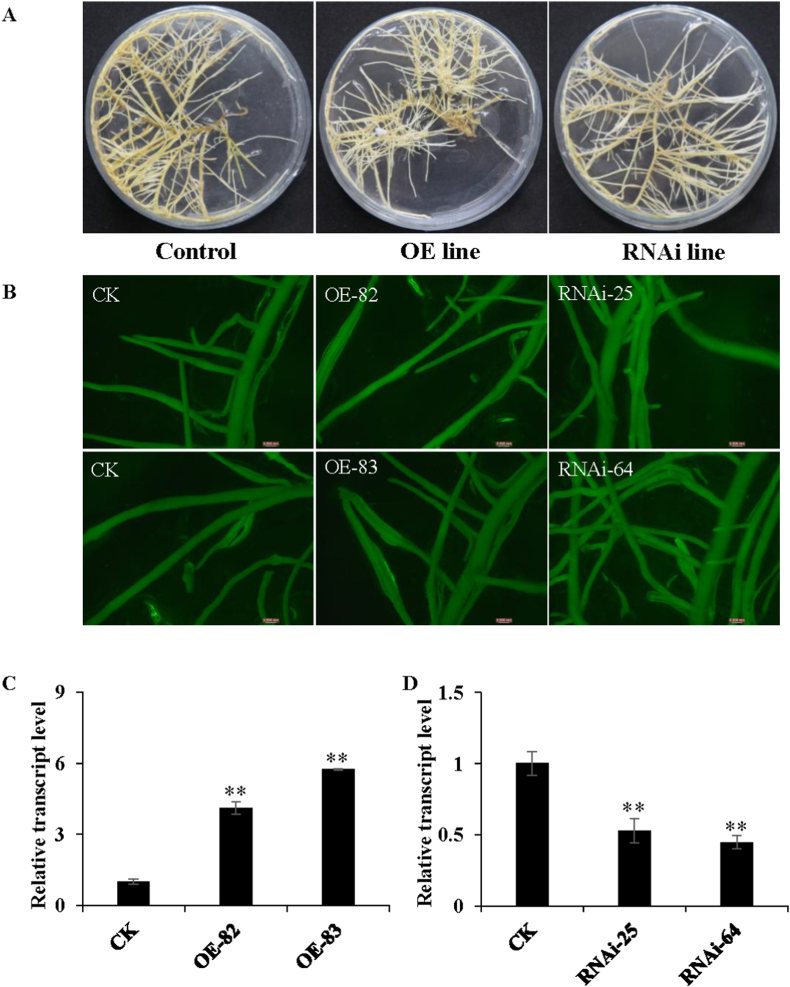
Over-expressing and silencing *GmCYP82D26* gene in soybean hairy roots. (A) Generation of soybean hairy roots over-expressing *GmCYP82D26* or silencing of *GmCYP82D2*6 by RNAi, with the vector control line. (B) The transgenic lines showing bright green fluorescence were detected under microscope. (C–D) The relative expression level of two representative overexpression lines (C) and two representative RNAi lines (D) as compared with that of the vector control line (as value of 1).

Hairy root system is an ideal system to efficiently investigate function of flavonoid pathway genes in soybean, we thus over-expressed *GmCYP82D26* in soybean hairy roots, as well as suppressed its expression by RNAi technology. The positive soybean hairy roots were selected by GFP signals under microscope owing to the presence of the GFP selection marker in both the vectors used for over-expression and RNAi (Fig. 3A and B). Moreover, the expression level of *GmCYP82D26* in both the over-expression lines and the RNAi lines were further confirmed by RT-qPCR. Among more than one hundred transgenic lines, two lines with relatively high expression level and another two lines with relatively low expression level were selected as representatives for metabolites analysis (Fig. 3C and D).

**Fig. 4:**
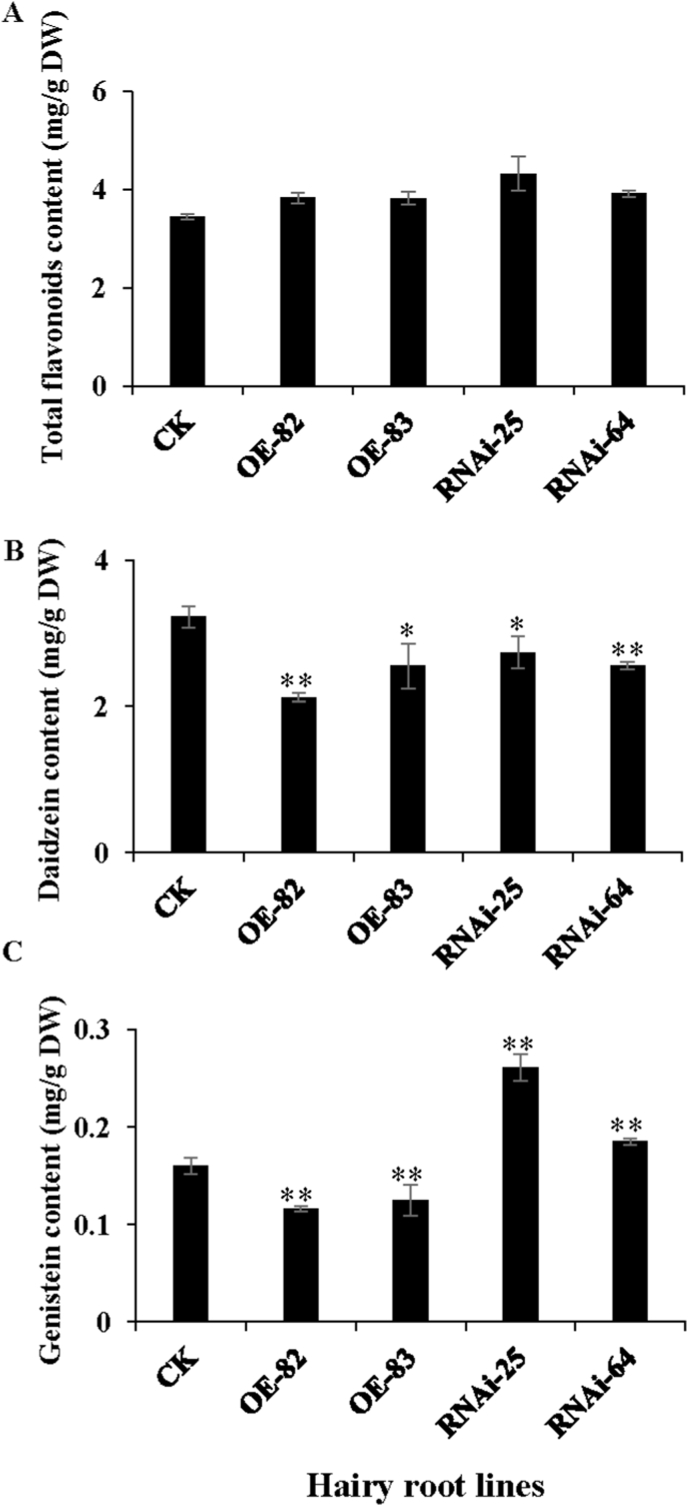
Analysis of total flavonoids, daidzein and genistein content of transgenic hairy roots over-expressing and silencing *GmCYP82D26*. (A) Content of total flavonoids in transgenic hairy roots of overexpressing and silencing *GmCYP82D26.* (B) Content of daidzein in transgenic hairy roots of overexpressing and silencing *GmCYP82D26* as measure by using UPLC; (C) Content of genistein in transgenic hairy roots of overexpressing and silencing *GmCYP82D26* as measure by using. Data are presented as mean ± SD, Student's *t*-test (n = 3, *P < 0.05, **P < 0.01).

We measured total flavonoid level in all transgenic lines, but no significant changes were found between transgenic lines or the vector control line (Fig. 4A). Furthermore, isoflavonoids composition and content were detected by using UPLC in both over-expression and RNAi hairy root lines (Fig. 4B and C, Fig. S7). It was shown that daidzein content in lines OE-82, OE-83, RNAi-25 and RNAi-64 were decreased to 2.12, 2.55, 2.74 and 2.56 mg/g DW as compared to 3.22 mg/g DW in the vector control line (Fig. 4B). In addition, genistein content in hairy root lines decreased to 0.12 and 0.13 mg/g DW in lines OE-82 and OE-83 as compared to 0.16 mg/g DW in the vector control (Fig. 4C), while genistein content increased significantly to 0.26 and 0.18 mg/g DW in lines RNAi-25 and RNAi-64 (Fig. 4C). Taken together, these data indicated that over-expression of *GmCYP82D26* led to decreased daidzein and genistein content in the hairy roots, but suppression of *GmCYP82D26* led to increased genistein content by reducing daidzein content.

### 3.5 Spatial expression pattern of *GmCYP82D26* gene in soybean

**Fig. 5:**
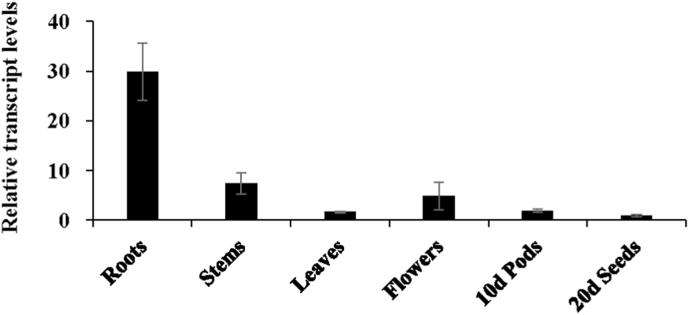
Spatial expression pattern of *GmCYP82D26* in soybean. Relative expression level of *GmCYP82D26* in roots, stems, leaves (three-week-old), flowers, pods (10 days after flowering) and seeds (20 days after flowering), as detected by RT-qPCR. The relative transcript levels of *GmCYP82D26* was determined by using the 2^−ΔΔCt^ method and normalized with *SUB3* gene as a reference. Transcript levels were expressed relative to that of seeds (20 days after flowering) that was set as 1.

By using RT-qPCR technique, we determined the relative expression level of *GmCYP82D26* gene in various tissues of soybean, including roots, stems, leaves, flowers of seedlings, as well as pods 10 days after flowering and seeds 20 days after flowering (Fig. 5). It was revealed that *GmCYP82D26* was preferentially expressed in roots, which was consistent with the above-mentioned transcriptome data as shown in Fig. 1A and previous results [36]. In addition, *GmCYP82D26* was also expressed in stems, leaves, flowers, young pods and seeds, but at much low level (Fig. 5).

### 3.6 GmCYP82D26 protein is localized in endoplasmic reticulum

**Fig. 6:**
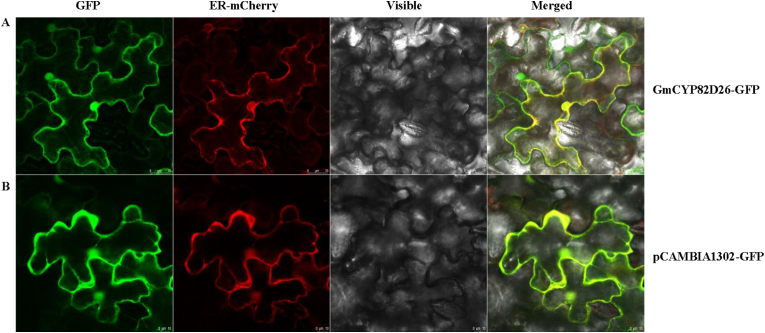
Subcellular localization of GmCYP82D26 in tobacco leaf epidermis. (A) Fluorescence signals of GmCYP82D26-GFP fusion protein. (B) Fluorescence signals of the empty vector pCAMBIA1302-GFP. The pCAMBIA1302 vector containing GmCYP82D26-GFP or GFP alone were transiently expressed in tobacco leaves, and the fluorescence signals of GFP (left), the fluorescence signals of mCherry (middle left), the signal in bright field (middle right), and the overlapping signals (right) were collected under confocal microscope 48 h later after infiltration. Bars = 25 μm.

To explore potential subcellular localization of GmCYP82D26, it was fused with GFP at C-terminal with the expression vector pCAMBIA1302. The resulting pCAMBIA1302 vector containing GmCYP82D26-GFP was transiently expressed in tobacco leaf epidermis by infiltration, together with the empty control vector. It was shown that the green fluorescence signals of the GmCYP82D26-GFP fusion protein overlapped with the mCherry fluorescence signal, which acts as an endoplasmic reticulum (ER) marker (Fig. 6A). Therefore, it is obvious that GmCYP82D26 is localized in endoplasmic reticulum. As the control, the fluorescence signal for pCAMBIA1302-GFP was distributed in the cytosol of tobacco epidemal cells (Fig. 6B).

### 3.7 Modeling and docking of GmCYP82D26 protein with naringenin

**Fig. 7:**
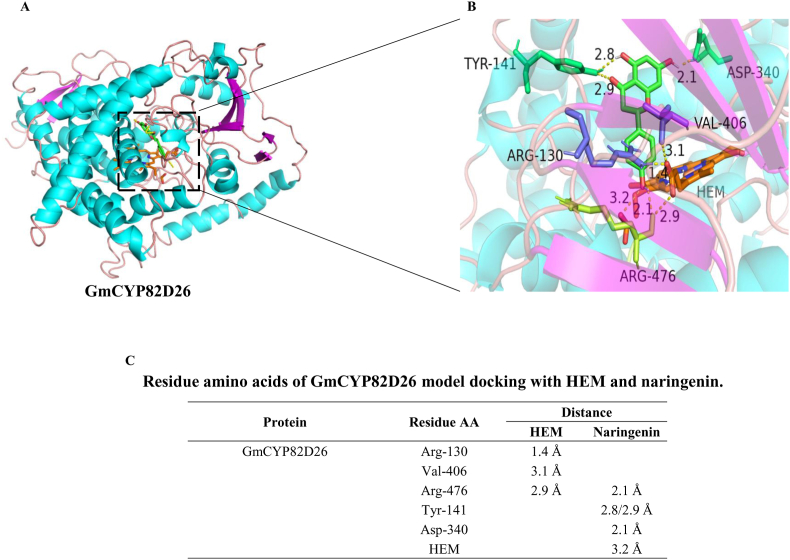
Structural modeling of GmCYP82D26 docking with HEM and naringenin. (A) the overall docking model: α-helix in cyan, β-sheet in fuchsia, and randon coil in darkorange. (B) the amino acid residues binding HEM and the naringenin: HEM is displayed in coral, naringenin is displayed in green and red; the amino acid residues binding HEM are shown in slateblue, the amino acid residues binding naringenin are shown in limegreen, and the amino acid residues binding both naringenin and HEM are shown in limon. (C) Amino acid residues of GmCYP82D26 model docking with HEM and naringenin.

To investigate potential catalytic mechanism of GmCYP82D26, its three-dimensional structure model was obtained through homology modeling with naringenin as substrate together with other five GmCYP82 proteins (Fig. 7 and Fig. S8). The docking data explained naringenin as ideal substrate for GmCYP82AD26. In the model, GmCYP82D26 bound with protoporphyrin iron (HEM) were docked with naringenin as shown with Autodock 4.2. In details, the amino acid residues Arg130, Val406 and Arg476 of GmCYP82AD26 could form hydrogen bonds with HEM, and the hydrogen bond distances are 1.4 Å, 3.1 Å and 2.9 Å, respectively. Tyr141 could form hydrogen bond with 5-OH of naringenin (2.8 Å) and C4 carbonyl of naringenin (2.9 Å). Asp340 could form hydrogen bond with 7-OH of naringenin (2.1 Å), Arg476 with 4′-OH of naringenin (2.1 Å), and HEM within GmCYP82D26 form hydrogen bond with 4′-OH of naringenin (3.2 Å) (Fig. 7).

Among the other five proteins, GmCYP82A2 and GmCYP82A4 could not form hydrogen bond with HEM, or with C4 carbonyl and 5-OH of naringenin. GmCYP82A3 could form hydrogen bond with HEM, but not with C4 carbonyl or 5-OH of naringenin (Figs. S8A, C, F). Although GmCYP82A23 could form hydrogen bond with C4 carbonyl and 5-OH of naringenin, but not with HEM (Figs. S8D and F). Even though GmCYP82C20 could form hydrogen bond with HEM, C4 carbonyl and 5-OH of naringenin as GmCYP82D26, but the number of amino acids (Phe-127, Leu325 and Ser-391) involving in forming hydrogen bond with HEM and naringenin was less for GmCYP82C20 than for GmCYP82D26 (Arg3, Val406, Arg476, Tyr141, and Asp340, Figs. S8E and F), suggesting that GmCYP82D26 might be more suitable and efficiency in catalyzing naringenin than the other five proteins.

## 4 Discussion

Soybeans is rich in isoflavones, which are precursors of phytoalexins with antibacterial and antiviral effects, and antiherbivore activities that protect plants from pathogen infections [48,49]. One of the most well-known soybean phytoalexins is prenylated pterocarpans derived from glyceollins and daidzein, which inhibited the growth of pathogens, such as *P. sojae* and *M. phaseolina* [50,51]. In addition, isoflavones can serve as stress-resistant mediators with antioxidant activity that help neutralize reactive oxygen species (ROS) induced by stressful conditions in plant defense systems [52]. Furthermore, isoflavones are secreted from roots to exert functions in rhizosphere plant-microbe interactions, such as daidzein and genistein [53]. In particular, daidzein was recently shown to be involved in the modulation of rhizosphere bacterial communities in soybean [5].

Several members of the CYP82 subfamily have been reported to be involved in flavone biosynthetic pathway. Therefore, in this study, we characterized all members of the CYP82 subfamily genes, attempting to identify potential new genes involved in unknown pathway of isoflavonoid biosynthesis in soybean. It was found that CYP82 underwent multiple duplication and form large cluster of homologous sequence with 24 members (Table S1 and Fig. S3). Analyses on phylogenetic relationship, protein sequence, expression profiles revealed that 6 candidate CYP82 subfamily genes (*GmCYP82A2*, *GmCYP82A3*, *GmCYP82A4*, *GmCYP82A23*, *GmCYP82C20* and *GmCYP82D26*) showed tissue-specific and inducible expression patterns as other isoflavonoid pathway genes with known functions (Fig. 1 and Tables S3 and S4).

Among these six recombinant soybean CYP82s tested for enzymatic activity, only GmCYP82D26 showed activity toward naringenin but not other nine representative flavonoid/isoflavonoid substrates (Fig. 2 and Table S6), which is different from other CYP82 proteins as reported from other plant species. ObCYP82D33 from sweet basil and MpCYP82D62 from mint were both flavone 6-hydroxylase (F6H), which could convert flavone genkwanin to 7-*O*-methylscutellarein. However, 7-*O*-methylation is a prerequisite for 6-hydroxylation by ObCYP82D33 and MpCYP82D62, which showed very low activity towards apigenin lacking a 7-methyl group [54]. SbCYP82D1.1 and SbCYP82D2 protein identified in *S. baicalensis* were flavone 6-hydroxylase (F6H) and flavone 8-hydroxylase (F8H), respectively. SbCYP82D1.1 had broad substrate specificity for flavones such as chrysin and apigenin, which was responsible for synthesis of both baicalein and scutellarein in roots and aerials parts of *S. baicalensis*, respectively. SbCYP82D2 can accept only chrysin as substrate to produce norwogonin with high substrate specificity, although minor 6-hydroxylation activity could also be detected [44,55]. Unlike these above-mentioned CYP82D family members, GmCYP82D26 did not exhibit either 6-hydroxylation or 8-hydroxylation activities towards apigenin.

In soybean, except for GmCYP82D26, the other five soybean CYP82 proteins did not exhibit any activity towards the tested flavonoid/isoflavonoid substrate although they could also be docked with naringenin (Fig. S8), and they may redundant genes during evolution. However, it could not be excluded that they may be involved in other pathways in soybean, as CYP82D protein were also reported to be involved in biosynthesis of alkaloids [56,57] and furnocoumarins [58], beyond flavonoid/isoflavonoid.

**Fig. 8:**
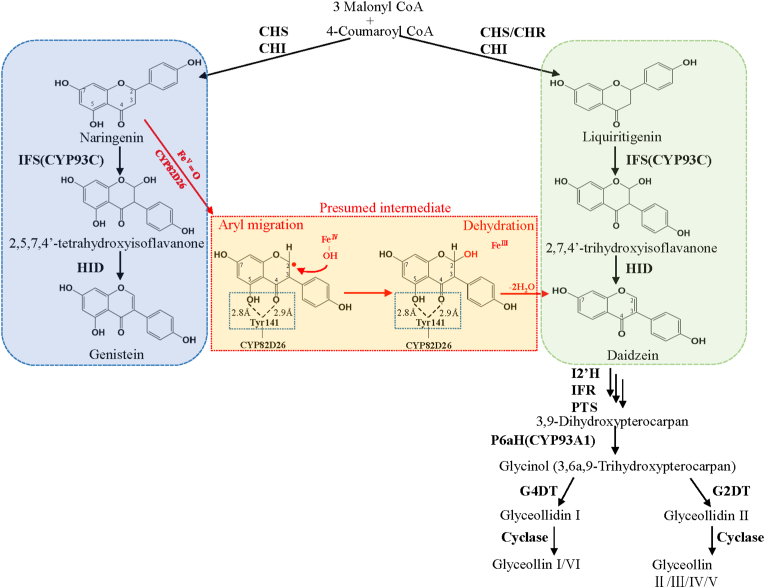
The diagram of CYP82D26 involved in the isoflavone biosynthetic pathway in soybeans. CHS, Chalcone synthase; CHR, Chalcone reductase; CHI, Chalcone isomerase; IFS, Isoflavone synthase; HID, 2-Hydroxyisoflavanone dehydratase; 5HID, 5-Hydroxyisoflavanone dehydratase; I2′H, Isoflavone 2′-hydroxylase; IFR, Isoflavone reductase; PTS, Pterocarpen synthase; P6αH, Pterocarpan 6α-hydroxylase; G2DT, (−)-Glycinol 2-dimethylallyltransferase; G4DT, (−)-Glycinol 4-dimethylallyltransferase. The red markers represent the newly identified functions of CYP82D26. The presumed intermediates and products corresponding to possible catalytic mechanisms were marked within red dotted box. The hydrogen bond formed by tyrosine 141 of CYP82D26 and naringenin is marked by the blue dotted box.

More importantly, it was found for the first time that GmCYP82D26 can catalyze naringenin to daidzein with both aryl migration and dehydration function (Fig. 8), which is different from all the other known CYP450s involve in flavonoid pathway. The function of CYP82D26 may be explained from the phylogenetic relationship viewpoint, as it was found that GmCYP82D26 may originate from the CYP93 family in the phylogenetic tree as previous report [36]. Among them, GmCYP93A1, GmCYP93C1v2 and GmCYP93C5 were involved in the biosynthesis of isoflavones, and GmCYP93B16 and GmCYP93B19 were involved in flavones [21,34,[59], [60], [61]]. It is possible that GmCYP82D26 gained both aryl migration function as CYP93 subfamily and gain dehydration function, due to gene duplication and mutation during evolution.

For the known CYP93 family proteins in soybean, GmCYP93A1 encodes pterocarpan 6α-hydroxylase (P6αH) [21], GmCYP93B encodes flavone synthase II (FNS II) [56] and CYP93C encoded isoflavone synthase (IFS) [34,62]. In term of the production of isoflavones with IFS, C-3 hydrogen of flavanone may be extracted by Fe^Ⅴ^ = O in the initial stage, followed by the migration of the side chain aryl (B-ring) from C-2 to C-3, and a hydroxyl is introduced at C-2 to produce C2-hydroxy isoflavanone by Fe^Ⅳ^-OH, which can be further dehydrated with hydrogen at C-3 by HID or automatically, and finally form isoflavones [1,35,61]. It is clear that CYP82D26 possesses aryl migration function as IFS, leading the B-ring to migrate from C2 to C3-position (Fig. 8) [1,63]. Soybean GmHID could catalyze 2,5,7,4′-tetrahydroxyisoflavanone, 2,7,4′-trihydroxyisoflavanone, and 2, 7-dihydroxy-4′-methoxyisoflavanone to produce genistein, daidzein and formononetin with relative activity 100%, 40% and 16%, respectively [55]. Gly78 and Gly79 of GmHID protein can each form a hydrogen bond with C4 carbonyl of substrate to form an oxyanion hole, and the activity of GmHID is greatly reduced when these two residues were mutated [55]. As shown from the protein structural modeling and docking, Tyr141 of CYP82D26 can form hydrogen bonds with both the C5 hydroxyl group and C4 carbonyl of naringenin (Figs. 3 and 8). Tyr141 of CYP82D26, like Gly78 and Gly79, is hydrophilic amino acid, which might be the key amino acid of CYP82D26 for the dehydration of C5 hydroxyl group of naringenin, which is similar as the dehydration reaction at C2 hydroxyl group of 2,5,7,4′-tetrahydroxyisoflavanone by HID (Fig. 8). Unlike GmHID, CYP82D26 was able to catalyze 2,5,7,4′-tetrahydroxyisoflavanone to produce daidzein instead of genistein, with additional aryl migration function. Furthermore, CYP82D26 catalyze the dehydration at both C2 and C5, with the loss of two water molecules (Fig. 8), which is different from GmHID with the loss of one water molecule. The function mechanism of CYP82D26 requires more evidence, nevertheless, the catalytic mechanism of CYP82D26 is more complex than other known CYP450 in isoflavonoid pathway in soybean.

GmCYP82D26 was localized in the endoplasmic reticulum when transiently expressed in tobacco epidermal cells (Fig. 6), which were the same as the other two P450 enzyme isoflavone synthase (IFS) and cinnamate 4-hydroxylase (C4H). IFS and C4H were tandem P450 enzymes anchored in the ER, and they interact with soluble enzymes of the phenylpropanoid and isoflavonoid pathways (chalcone synthase, chalcone reductase and chalcone isomerase) [64]. These results suggested that the existence of metabolon in soybean isoflavonoid biosynthesis.

*GmCYP82D26* showed the highest expression levels in roots as verified by both transcriptome and qRT-PCR analyses (Figs. 1A and 5 and Table S2). Meanwhile, *GmCYP82D26* was up-regulated upon *P. sojae* treatment (Fig. 1B and Table S3). Furthermore, *in vitro* and *in vivo* assays confirmed that GmCYP82D26 was involved in daidzein biosynthesis. The contents of daidzein in the hairy roots silencing *GmCYP82D26* were decreased compared with the control (Fig. 4B). The previous study showed that the main isoflavonoid compounds were daidzein and its derivatives in roots of soybean [65]. All above evidence confirmed that GmCYP82D26 is responsible for the biosynthesis of daidzein from naringenin in soybean roots. CYP82D26 thus provide an alternative branch pathway for the production of daidzein in soybean. As genistein content in the transgenic hairy roots of silencing *GmCYP82D26* was increased but daidzein was decrease, indicating GmCYP82D26 bridge the two branch pathways and balance the content of daidzein and genistein in soybean. Therefore, *GmCYP82D26* could be utilized for the conversion of naringenin to daidzein by using one-step synthetic biology approach.

## CRediT authorship contribution statement

**Yaying Xia:** Methodology, Investigation, and, Writing – original draft. **Chunfeng He:** Methodology, and, Investigation. **Su Yan:** Methodology, and, Investigation. **Jinyue Liu:** Investigation. **Haijun Huang:** Investigation. **Xue Li:** Investigation. **Qian Su:** Investigation. **Wenbo Jiang:** Methodology. **Yongzhen Pang:** Conceptualization, Resources, Writing – review & editing, and, Supervision.

## Declaration of competing interest

The authors declare that they have no known competing financial interests or personal relationships that could have appeared to influence the work reported in this paper.

## References

[1] Dixon R.A., Barton S.D., Nakanishi K., Meth-Cohn O. (1999). Comprehensive natural products chemistry.

[2] Dixon R.A., Steele C.L. (1999). Flavonoids and isoflavonoids - a gold mine for metabolic engineering. Trends Plant Sci.

[3] Dixon R.A., Sumner L.W. (2003). Legume natural products: understanding and manipulating complex pathways for human and animal health. Plant Physiol.

[4] Morris P.F., Bone E., Tyler B.M. (1998). Chemotropic and contact responses of *phytophthora sojae* hyphae to soybean isoflavonoids and artificial substrates. Plant Physiol.

[5] Okutani F., Hamamoto S., Aoki Y., Nakayasu M., Nihei N., Nishimura T. (2020). Rhizosphere modelling reveals spatiotemporal distribution of daidzein shaping soybean rhizosphere bacterial community. Plant Cell Environ.

[6] Dastmalchi M., Dhaubhadel S. (2015). Proteomic insights into synthesis of isoflavonoids in soybean seeds. Proteomics.

[7] Miadoková E. (2009). Isoflavonoids - an overview of their biological activities and potential health benefits. Interdiscipl Toxicol.

[8] Howes L.G., Howes J.B., Knight D.C. (2006). Isoflavone therapy for menopausal flushes: a systematic review and meta-analysis. Maturitas.

[9] Potter S.M., Baum J.A., Teng H., Stillman R.J., Shay N.F., Erdman J.W. (1998). Soy protein and isoflavones: their effects on blood lipids and bone density in postmenopausal women. Am J Clin Nutr.

[10] Strom B.L., Schinnar R., Ziegle E.E., Barhard K.T. (2001). Exposure to soy-based formula in infancy and endochinological and reproductive outcomes in young adulthood. J Am Med Dir Assoc.

[11] Walker H.A., Dean T.S., Sanders T.A.B., Jackson G., Ritter J.M., Chowienczyk P.J. (2001). The phytoestrogen genistein produces acute nitric oxide–dependent dilation of human forearm vasculature with similar potency to 17 β-estradiol. Circulation.

[12] Lamartiniere C.A. (2000). Protection against breast cancer with genistein: a component of soy. Am J Clin Nutr.

[13] Xu L., Ding Y., Catalona W.J., Yang X.J., Anderson W.F., Jovanovic B. (2009). MEK4 function, genistein treatment, and invasion of human prostate cancer cells. J Natl Cancer Inst.

[14] Messina M. (2014). Soy foods, isoflavones, and the health of postmenopausal women. Am J Clin Nutr.

[15] Hassan S.M., El-Shemy H.A. (2013). Soybean, nutrition and health.

[16] Wang H., Murphy P.A. (1994). Isoflavone content in commercial soybean foods. J Agric Food Chem.

[17] Austin M.B., Noel J.P. (2003). The chalcone synthase superfamily of type III polyketide synthases. Nat Prod Rep.

[18] Grotewold E. (2006). The genetics and biochemistry of floral pigments. Annu Rev Phytopathol.

[19] Mona M.C., Lamb C.J. (1987). Chalcone isomerase cDNA cloning and mRNA induction by fungal elicitor, wounding and infection. EMBO J.

[20] Jung W., Yu O., Lau S.M.C., O'Keefe D.P., Odell J., Fader G. (2000). Identification and expression of isoflavone synthase, the key enzyme for biosynthesis of isoflavones in legumes. Nat Biotechnol.

[21] Schopfera C.R., Kochs G., Lottspeich F., Ebel J. (1998). Molecular characterization and functional expression of dihydroxypterocarpan 6a-hydroxylase, an enzyme specific for pterocarpanoid phytoalexin biosynthesis in soybean (*Glycine max* L.). FEBS (Fed Eur Biochem Soc) Lett.

[22] Nwachukwu I.D., Luciano F.B., Udenigwe C.C. (2013). The inducible soybean glyceollin phytoalexins with multifunctional health promoting properties. Food Res Int.

[23] Duffy C., Perez K., Partridge A. (2007). Implications of phytoestrogen intake for breast cancer. CA A Cancer J Clin.

[24] Yoneyama K., Akashi T., Aoki T. (2016). Molecular characterization of soybean pterocarpan 2-dimethylallyltransferase in glyceollin biosynthesis: local gene and whole-genome duplications of prenyltransferase genes led to the structural diversity of soybean prenylated isoflavonoids. Plant Cell Physiol.

[25] Akashi T., Sasaki K., Aoki T., Ayabe S., Yazaki K. (2009). Molecular cloning and characterization of a cdna for pterocarpan 4-dimethylallyltransferase catalyzing the key prenylation step in the biosynthesis of glyceollin, a soybean phytoalexin. Plant Physiol.

[26] Welle R., Grisebach H. (1988). Induction of phytoalexin synthesis in soybean: enzymatic cyclization of prenylated pterocarpans to glyceollin isomers. Arch Biochem Biophys.

[27] Bak S., Beisson F., Bishop G., Hamberger B., Hofer R., Paquette S. (2011). The Arabidopsis book.

[28] Artigot M., Baes M., Daydé J., Berger M. (2013). Expression of flavonoid 6-hydroxylase candidate genes in normal and mutant soybean genotypes for glycitein content. Mol Biol Rep.

[29] Holton T.A., Brugliera F., Lester D.R., Tanaka Y., Hyland C.D., Menting J.G.T. (1993). Cloning and expression of cytochrome P450 genes controlling flower colour. Nature.

[30] Akashi T., Aoki T., Ayabe S. (1998). CYP81E1, a cytochrome P450 cDNA of licorice (*Glycyrrhiza echinata* L.), encodes isoflavone 2'-hydroxylase. Biochem Biophys Res Commun.

[31] Liu C.J., Huhman D., Sumner L.W., Dixon R.A. (2003). Regiospecific hydroxylation of isoflavones by cytochrome P450 81E enzymes from *Medicago truncatula*. Plant J..

[32] Akashi T., Aoki T., Ayabe S. (1998). Identification of a cytochrome P450 cDNA encoding (2S)-flavanone 2-hydroxylase of licorice (*Glycyrrhiza echinata* L.; Fabaceae) which represents licodione synthase and flavone synthase II. FEBS (Fed Eur Biochem Soc) Lett.

[33] Du H., Ran F., Dong H.L., Wen J., Li J.N., Liang Z. (2016). Genome-wide analysis, classification, evolution, and expression analysis of the cytochrome P450 93 family in land plants. PLoS One.

[34] Steele C.L., Gijzen M., Qutob D., Dixon R.A. (1999). Molecular characterization of the enzyme catalyzing the aryl migration reaction of isoflavonoid biosynthesis in soybean. Arch Biochem Biophys.

[35] Akashi T., Aoki T., Ayabe S. (1999). Cloning and functional expression of a cytochrome P450 cDNA encoding 2-hydroxyisoflavanone synthase involved in biosynthesis of the isoflavonoid skeleton in licorice. Plant Physiol.

[36] Guttikonda S.K., Trupti J., Bisht N.C., Chen H., An Y.Q., Pandey S. (2010). Whole genome co-expression analysis of soybean cytochrome P450 genes identifies nodulation-specific P450 monooxygenases. BMC Plant Biol.

[37] Kumar S., Stecher G., Li M., Knyaz C., Tamura K. (2018). Mega X: molecular evolutionary genetics analysis across computing platforms. Mol Biol Evol.

[38] Jahan M.A., Harris B., Lowery M., Infante A.M., Percifield R.J., Kovinich N. (2020). Glyceollin transcription factor GmMYB29A2 regulates soybean resistance to *Phytophthora sojae*. Plant Physiol.

[39] Yang J., Zhang Y. (2015). I-TASSER server: new development for protein structure and function predictions. Nucleic Acids Res.

[40] Trott O., Olson A.J. (2009). AutoDock Vina: improving the speed and accuracy of docking with a new scoring function, efficient optimization, and multithreading. J Comput Chem.

[41] Kumari V., Kumar V., Bhall T.C. (2015). Functional interpretation and structural insights of *Arabidopsis lyrata* cytochrome P450 CYP71A13 involved in auxin synthesis. Bioinformation.

[42] Wang Z., Li J., Chen S., Heng Y., Chen Z., Yang J. (2017). Poaceae-specific MS1 encodes a phospholipid-binding protein for male fertility in bread wheat. Proc Natl Acad Sci USA.

[43] Livak K.J., Schmittgen T.D. (2001). Analysis of relative gene expression data using real-time quantitative PCR and the 2^−ΔΔCT^ Method. Methods.

[44] Zhao Q., Cui M.Y., Levsh O., Yang D., Liu J., Li J. (2018). Two CYP82D enzymes function as flavone hydroxylases in the biosynthesis of root-specific 4’-deoxyflavones in *Scutellaria baicalensis*. Mol Plant.

[45] Hasemann C.A., Kurumbail R.G., Boddupalli S.S., Peterson J.A., Deisenhofer J. (1995). Structure and function of cytochromes P450: a comparative analysis of three crystal structures. Structure.

[46] Aoki K., Ogata Y., Shibata D. (2007). Approaches for extracting practical information from gene co-expression networks in plant biology. Plant Cell Physiol.

[47] Pompon D., Louerat B., Bronine A., Urba P. (1996). Yeast expression of animal and plant P450s in optimized redox environments. Methods Enzymol.

[48] Singh S., Kaur I., Kariyat R. (2021). The multifunctional roles of polyphenols in plant-herbivore interactions. Int J Mol Sci.

[49] Dixon R.A. (2001). Natural products and plant disease resistance. Nature.

[50] Sukumaran A., McDowell T., Chen L., Renaud J., Dhaubhadel S. (2018). Isoflavonoid-specific prenyltransferase gene family in soybean: GmPT01, a pterocarpan 2-dimethylallyltransferase involved in glyceollin biosynthesis. Plant J.

[51] Lygin A.V., Hill C.B., Zernova O.V., Crull L., Widholm J.M., Hartman G.L. (2010). Response of soybean pathogens to glyceollin. Phytopathology.

[52] Kim I.S. (2021). Current perspectives on the beneficial effects of soybean isoflavones and their metabolites for humans. Antioxidants.

[53] Toyofuku M., Okutani F., Nakayasu M., Hamamoto S., Takase H., Yazaki K. (2021). Enhancement of developmentally regulated daidzein secretion from soybean roots in field conditions as compared with hydroponic culture. Biosci, Biotechnol, Biochem.

[54] Berim A., Gang D.R. (2013). The roles of a tlavone-6-hydroxylase and 7-*O*-demethylation in the flavone biosynthetic network of *Sweet Basil*. J Biol Chem.

[55] Gao R., Lou Q., Hao L., Qi G., Tian Y., Pu X., He C., Wang Y., Xu W., Xu Z., Song J. (2022). Comparative genomics reveal the convergent evolution of CYP82D and CYP706X members related to flavone biosynthesis in Lamiaceae and Asteraceae. Plant J.

[56] Dang T.T., Facchini P.J. (2014). CYP82Y1 is N-methylcanadine 1-hydroxylase, a key noscapine biosynthetic enzyme in opium poppy. J Biol Chem.

[57] Takemura T., Ikezawa N., Iwasa K., Sato F. (2013). Molecular cloning and characterization of a cytochrome P450 in sanguinarine biosynthesis from *Eschscholzia californica* cells. Phytochemistry.

[58] Limones-Mendez M., Dugrand-Judek A., Villard C., Coqueret V., Froelicher Y., Bourgaud F., Olry A., Hehn A. (2020). Convergent evolution leading to the appearance of furanocoumarins in citrus plants. Plant Sci.

[59] Akashi T., Aoki T., Ayabe S. (2005). Molecular and biochemical characterization of 2-hydroxyisoflavanone dehydratase. Involvement of carboxylesterase-like proteins in leguminous isoflavone biosynthesis. Plant Physiol.

[60] Fliegmann J., Furtwängler K., Malterer G., Cantarello C., Schüler G., Ebel J. (2010). Flavone synthase II (CYP93B16) from soybean (*Glycine max* L.). Phytochemistry.

[61] Sawada Y., Kinoshita K., Akashi T., Aoki T., Ayabe S. (2002). Key amino acid residues required for aryl migration catalysed by the cytochrome P450 2-hydroxyisoflavanone synthase. Plant J.

[62] Ayabe S., Akashi T., Aoki T. (2002). Cloning of cDNAs encoding P450s in the flavonoid/isoflavonoid pathway from elicited leguminous cell cultures. Methods Enzymol.

[63] Mansuy D., Mansuy D. (1998). The great diversity of reactions catalyzed by cytochromes P450.

[64] Dastmalchi M., Bernards M.A., Dhaubhadel S. (2016). Twin anchors of the soybean isoflavonoid metabolon: evidence for tethering of the complex to the endoplasmic reticulum by IFS and C4H. Plant J.

[65] Graham T.L., Kim J.E., Graham M.Y. (1990). Role of constitutive isoflavone conjugates in the accumulation of glyceollin in soybean infected with Phytophthora megasperma. Mol Plant Microbe Interact.

