# Supplementary material for: New dual functional CYP450 gene involves in isoflavone biosynthesis in Glycine max L
Source: Synth Syst Biotechnol. 2023 Jan 6;8(1):157–67. doi: 10.1016/j.synbio.2023.01.002 (PMC9860299; doi:10.1016/j.synbio.2023.01.002)
Supplement: Multimedia component 1 [file mmc1.docx]

Supplementary materials

Table S1. The CYP82 subfamily genes and the corresponding plant species used in this study.

| Gene name | Gene ID | *Species* |
| --- | --- | --- |
| *GmCYP93C5* | Glyma.07G202300 | *Glycine max* |
| *GmCYP82A2* | Glyma.13G285300 | *Glycine max* |
| *GmCYP82A3* | Glyma.13G068800 | *Glycine max* |
| *GmCYP82A4* | Glyma.01G135200 | *Glycine max* |
| *GmCYP82A18* | Glyma.19G015300 | *Glycine max* |
| *GmCYP82A19* | Glyma.19G015200 | *Glycine max* |
| *GmCYP82A20* | Glyma.19g015000 | *Glycine max* |
| *GmCYP82A22* | Glyma.19g014700 | *Glycine max* |
| *GmCYP82A23* | Glyma.19G014600 | *Glycine max* |
| *GmCYP82A24* | Glyma.15g203500 | *Glycine max* |
| *GmCYP82A25* | Glyma.02g078800 | *Glycine max* |
| *GmCYP82A26* | Glyma.13g068500 | *Glycine max* |
| *GmCYP82C1* | Glyma.11g060100 | *Glycine max* |
| *GmCYP82C18* | Glyma.11g060200 | *Glycine max* |
| *GmCYP82C20* | Glyma.01G181800 | *Glycine max* |
| *GmCYP82C21* | Glyma.01g181900 | *Glycine max* |
| *GmCYP82D25* | Glyma.04g035500 | *Glycine max* |
| *GmCYP82D26* | Glyma.06G035300 | *Glycine max* |
| *GmCYP82D27* | Glyma.06g035200 | *Glycine max* |
| *GmCYP82D28* | Glyma.06G035400 | *Glycine max* |
| *GmCYP82D29* | Glyma.04g035600 | *Glycine max* |
| *GmCYP82J3* | Glyma.16g090000 | *Glycine max* |
| *GmCYP82L4* | Glyma.16g089700 | *Glycine max* |
| *GmCYP82L5* | Glyma.16g089900 | *Glycine max* |
| *GmCYP82L6* | Glyma.03g085000 | *Glycine max* |
| *AtCYP82C2* | AT4G31970.1 | *Arabidopsis thaliana* |
| *AtCYP82C3* | AT4G31950.1 | *Arabidopsis thaliana* |
| *AtCYP82C4* | AT4G31940.1 | *Arabidopsis thaliana* |
| *AtCYP82F1* | AT2G25160.1 | *Arabidopsis thaliana* |
| *AtCYP82G1* | AT3G25180.1 | *Arabidopsis thaliana* |
| *SbCYP82D1.1* | A0A286RS91 | *Scutellaria baicalensis* |
| *SbCYP82D1.2* | A0A286RS89 | *Scutellaria baicalensis* |
| *SbCYP82D2* | A0A286RS92 | *Scutellaria baicalensis* |
| *GhCYP82D1* | A0A0A0QDR9 | *Gossypium hirsutum* |
| *GhCYP82D2* | A0A0A0QBB1 | *Gossypium hirsutum* |
| *GhCYP82D3* | A0A0A0QG42 | *Gossypium hirsutum* |
| *NtCYP82E4v1* | ABA07805.1 | *Nicotiana tabacum* |
| *ObCYP82D33* | AGF30364.1 | *Ocimum basilicum* |
| *EcCYP82B1* | AAC39454.1 | *Eschscholzia californica* |
| *EcCYP82N2v2* | BAK20464.1 | *Eschscholzia californica* |
| *PsCYP82N3* | KC154002 | *Papaver somniferum* |
| *PsCYP82N4* | KC154003 | *Papaver somniferum* |
| *PsCYP82Y1* | JQ659005 | *Papaver somniferum* |
| *MpCYP82D62* | AGF30366.1 | *Mentha × piperita* |

Table S2. Expression level of soybean *CYP82* and isoflavone pathway genes in different tissues of soybean.

| Gene name | root | nodule | young  leaf | flower | one cm pod | pod shell 10DAF | pod shell 14DAF | seed 10DAF | seed 14DAF | seed 21DAF | seed 25DAF | seed 28DAF | seed 35DAF | seed 42DAF |
| --- | --- | --- | --- | --- | --- | --- | --- | --- | --- | --- | --- | --- | --- | --- |
| *GmHIDH* | 132 | 44 | 18 | 12 | 32 | 29 | 26 | 3 | 4 | 5 | 5 | 2 | 3 | 3 |
| *GmCYP93C5(IFS1)* | 129 | 23 | 0 | 3 | 0 | 0 | 0 | 0 | 2 | 3 | 13 | 14 | 37 | 54 |
| *GmCYP93C1v2(IFS2)* | 328 | 53 | 8 | 0 | 1 | 0 | 1 | 0 | 2 | 4 | 13 | 13 | 32 | 37 |
| *GmCYP93A1(P6αH)* | 4 | 7 | 0 | 1 | 0 | 0 | 0 | 0 | 0 | 0 | 0 | 0 | 0 | 0 |
| *GmG4DT* | 6 | 2 | 0 | 0 | 0 | 0 | 0 | 0 | 0 | 0 | 0 | 0 | 0 | 0 |
| *GmG2DT-1* | 2 | 1 | 0 | 2 | 0 | 0 | 0 | 0 | 0 | 0 | 0 | 0 | 0 | 0 |
| *GmG2DT-2* | 2 | 1 | 0 | 4 | 1 | 1 | 1 | 0 | 0 | 0 | 0 | 0 | 0 | 0 |
| *GmCYP82A2* | 2 | 1 | 0 | 7 | 2 | 1 | 1 | 0 | 0 | 0 | 0 | 0 | 1 | 1 |
| *GmCYP82A3* | 1 | 0 | 0 | 0 | 0 | 0 | 0 | 0 | 0 | 0 | 0 | 0 | 0 | 0 |
| *GmCYP82A4* | 1 | 1 | 0 | 10 | 0 | 0 | 0 | 0 | 0 | 0 | 0 | 0 | 0 | 0 |
| *GmCYP82A18* | 0 | 0 | 1 | 70 | 2 | 2 | 3 | 0 | 0 | 0 | 0 | 0 | 0 | 0 |
| *GmCYP82A19* | 0 | 0 | 0 | 81 | 1 | 1 | 0 | 0 | 0 | 0 | 0 | 0 | 0 | 0 |
| *GmCYP82A20* | 0 | 0 | 0 | 2 | 0 | 0 | 0 | 0 | 0 | 0 | 0 | 0 | 0 | 0 |
| *GmCYP82A22* | 4 | 1 | 0 | 0 | 0 | 0 | 0 | 0 | 0 | 0 | 0 | 0 | 0 | 0 |
| *GmCYP82A23* | 6 | 0 | 0 | 0 | 0 | 0 | 0 | 0 | 0 | 0 | 0 | 0 | 0 | 0 |
| *GmCYP82A24* | 4 | 0 | 0 | 2 | 0 | 0 | 0 | 0 | 0 | 0 | 0 | 0 | 0 | 0 |
| *GmCYP82A25* | 0 | 0 | 0 | 1 | 0 | 0 | 0 | 2 | 3 | 3 | 1 | 0 | 0 | 0 |
| *GmCYP82A26* | 0 | 0 | 1 | 10 | 1 | 1 | 1 | 1 | 2 | 2 | 2 | 0 | 0 | 0 |
| *GmCYP82C1* | 0 | 0 | 3 | 5 | 6 | 4 | 3 | 2 | 7 | 4 | 3 | 2 | 5 | 2 |
| *GmCYP82C18* | 1 | 0 | 1 | 4 | 1 | 0 | 0 | 0 | 0 | 0 | 1 | 0 | 2 | 1 |
| *GmCYP82C20* | 10 | 3 | 0 | 1 | 0 | 0 | 0 | 0 | 0 | 0 | 0 | 0 | 0 | 0 |
| *GmCYP82C21* | 0 | 0 | 0 | 4 | 1 | 0 | 0 | 1 | 0 | 0 | 1 | 1 | 2 | 2 |
| *GmCYP82D25* | 2 | 1 | 1 | 4 | 6 | 4 | 3 | 2 | 2 | 3 | 3 | 2 | 2 | 1 |
| *GmCYP82D26* | 31 | 2 | 1 | 1 | 1 | 3 | 1 | 0 | 0 | 0 | 0 | 0 | 0 | 0 |
| *GmCYP82D27* | 0 | 0 | 2 | 7 | 0 | 0 | 0 | 0 | 0 | 0 | 0 | 0 | 0 | 0 |
| *GmCYP82D28* | 1 | 2 | 3 | 4 | 2 | 2 | 1 | 1 | 1 | 2 | 1 | 1 | 1 | 1 |
| *GmCYP82D29* | 83 | 0 | 2 | 0 | 0 | 0 | 0 | 0 | 0 | 0 | 0 | 0 | 0 | 0 |
| *GmCYP82J3* | 2 | 1 | 1 | 3 | 3 | 4 | 3 | 0 | 0 | 0 | 0 | 0 | 0 | 0 |
| *GmCYP82L4* | 0 | 0 | 0 | 4 | 0 | 0 | 0 | 0 | 0 | 0 | 0 | 0 | 0 | 0 |
| *GmCYP82L5* | 0 | 0 | 0 | 0 | 0 | 0 | 0 | 0 | 0 | 0 | 0 | 0 | 0 | 0 |
| *GmCYP82L6* | 0 | 0 | 0 | 1 | 0 | 0 | 0 | 0 | 0 | 0 | 0 | 0 | 0 | 0 |

Data were retrieved from soybase website (https://soybase.org/soyseq/). DAF: day after flowering.

**Table S3. Soybean CYP82 subfamily genes that were up-regulated by *P. sojae* infection*.***

| Gene name | high resistance | | | | | | | | Moderate resistance | | | | low resistance | | | |
| --- | --- | --- | --- | --- | --- | --- | --- | --- | --- | --- | --- | --- | --- | --- | --- | --- |
|  | Athow | | Conrad | | General | | V710370 | | PI291327 | | Williams | | OX20-8 | | Sloan | |
|  | 72h | 120h | 72h | 120h | 72h | 120h | 72h | 120h | 72h | 120h | 72h | 120h | 72h | 120h | 72h | 120h |
| *GmHIDH* | 3.4 | 4.0 | 3.9 | 5.3 | 4.5 | 5.7 | 4.4 | 7.7 | 6.7 | 9.7 | 4.8 | 6.3 | 4.1 | 13.2 | 2.1 | 8.7 |
| *GmCYP93A1(P6αH)* | 4.1 | 3.2 | 4.6 | 4.2 | 3.7 | 5.0 | 3.2 | 3.8 | 6.9 | 6.2 | 5.5 | 5.2 | 5.7 | 7.5 | 3.2 | 7.1 |
| *GmCYP93C5(IFS1)* | 1.5 | 1.7 | 1.5 | 2.0 | 2.2 | 3.0 | 2.5 | 3.8 | 3.4 | 4.6 | 1.6 | 2.1 | 1.9 | 3.7 | 1.0 | 2.6 |
| *GmCYP93C1v2(IFS2)* | 6.1 | 5.9 | 5.2 | 6.0 | 7.2 | 7.5 | 7.1 | 11.4 | 9.4 | 13.9 | 8.2 | 11.0 | 6.7 | 23.1 | 2.8 | 11.6 |
| *GmG4DT* | 10.0 | 14.0 | 15.3 | 10.8 | 12.3 | 23.5 | 10.0 | 18.6 | 18.2 | 35.7 | 15.3 | 19.9 | 20.8 | 29.5 | 7.2 | 26.0 |
| *GmG2DT-1* | 1.6 | 2.2 | 2.0 | 2.5 | 1.6 | 2.1 | 1.8 | 2.2 | 1.7 | 2.1 | 2.1 | 2.0 | 1.7 | 3.2 | 1.4 | 2.8 |
| *GmG2DT-2* | 6.8 | 12.3 | 8.3 | 13.1 | 8.9 | 16.6 | 9.3 | 10.0 | 21.0 | 54.2 | 16.4 | 38.8 | 8.3 | 46.2 | 4.6 | 29.6 |
| *GmCYP82A2* | 10.1 | 16.4 | 12.1 | 19.1 | 13.8 | 23.7 | 11.8 | 25.8 | 24.1 | 78.7 | 17.7 | 39.3 | 15.0 | 102.2 | 5.5 | 45.2 |
| *GmCYP82A3* | 1.0 | 1.2 | 1.4 | 1.8 | 1.6 | 2.3 | 1.2 | 1.3 | 1.6 | 1.5 | 1.3 | 1.5 | 1.8 | 2.7 | 1.1 | 1.4 |
| *GmCYP82A4* | 55.7 | 68.3 | 84.8 | 35.2 | 68.5 | 121.6 | 50.9 | 74.7 | 213.9 | 218.4 | 111.6 | 217.2 | 112.1 | 463.6 | 19.2 | 270.5 |
| *GmCYP82D26* | 2.2 | 2.2 | 2.3 | 2.1 | 2.7 | 2.4 | 2.8 | 4.0 | 3.6 | 5.4 | 2.4 | 3.1 | 2.0 | 4.9 | 1.8 | 4.9 |

Data were retrieved from affymetrix soybean genome array (GDS3242). The fold change of was calculated by comparing gene expression level in *P. sojae* treated samples with the mock control.

Table S4. Soybean CYP82 subfamily genes and isoflavone pathway genes that were up-regulated by WGE in W82 hairy roots.

| Gene name | W82 Hairy Roots | | |
| --- | --- | --- | --- |
|  | Fold Change | log2 Fold Change | p-value |
| *GmHIDH* | 1.537 | 0.62 | 1.1E+205 |
| *GmCYP93A1* | 2.378 | 1.25 | 5.99E-25 |
| *GmCYP93C5* | 1.234 | 0.303 | 1.75E-03 |
| *GmCYP93C1v2* | 3.082 | 1.624 | 2.22E-25 |
| *GmG4DT* | 5.696 | 2.51 | 2.3E+299 |
| *GmCYP82A2* | 12.606 | 3.656 | 4.21E-58 |
| *GmCYP82A3* | 3.829 | 1.937 | 3.07E-18 |
| *GmCYP82A4* | 26.927 | 4.751 | 1.28E-60 |
| *GmCYP82A23* | 1.709 | 0.773 | 1.85E-06 |
| *GmCYP82C20* | 1.350 | 0.433 | 0.00369 |

Data were retrieved from transcriptome database (GSE131686) (Jahan et al., 2020)

Table S5. The primer sequences used in the present study.

| Gene | Primer Name | Primer Sequence (5’ to 3’) | Purpose | Product Length(bp) |
| --- | --- | --- | --- | --- |
| *SUB-3* | SUB-3 qRT-F | GTGTAATGTTGGATGTGTTCCC | qRT-PCR | 100 |
|  | SUB-3 qRT-R | ACACAATTGAGTTCAACACAAACCG |  |  |
| *GmCYP82A2* | 82A2-BamH I-F | cgGGATCCATGGAGTTAGTTCTAAACAGCAC | Yeast expression | 1569 |
|  | 82A2-Sma I-R | tccCCCGGGTTAGATACTTTCATAACAACTA |  |  |
| *GmCYP82A3* | 82A3-BamH I-F | cgGGATCCATGGACCTTCTCCTAAATTGC | Yeast expression | 1584 |
|  | 82A3-Kpn I-R | ggGGTACCTTATAAAGTTTCATAATAGTTGGGAG |  |  |
| *GmCYP82A4* | 82A4-Sma I-F | tccCCCGGGATGGAATTAGTTCTACATTTCCT | Yeast expression | 1581 |
|  | 82A4-Kpn I-R | ggGGTACCTCACATACTTTTGTAACAACTT |  | |
| *GmCYP82A23* | 82A23-BamH I-F | cgGGATCCATGGACCTTCTCCTAAATTGCAT | Yeast expression | 1584 |
|  | 82A23-Sac I-R | tccGAGCTCTTACAAAGTTTCATAATAATTG |  | |
| *GmCYP82C20* | 82C20-BamH I-F | cgGGATCCATGGTCATGGTCATGGATGT | Yeast expression | 1593 |
|  | 82C20-Sma I-R | tccCCCGGGTTAGTTCTCATAAAGCTTTGTATC |  | |
| *GmCYP82D26* | 82D26-Sma I-F | tccCCCGGGATGATAATCCATTATCAAAATC | Yeast expression | 1626 |
|  | 82D26-Sac I-R | tccGAGCTCTTAAATTTCATCATAAATATGACC |  |  |
| *GmCYP82D26* | 82D26-OE-F | CACCATGATAATCCATTATCAAAATC | Over-expression | 1640 |
|  | 82D26-OE-R | TTAAATTTCATCATAAATATGACC |  |  |
|  | 82D26-Nco I-F | tatCCATGGTAATGATAATCCATTATCAAAATCA | Subcellular location | 1623 |
|  | 82D26-Bgl II-R | TATAGATCTCCAATTTCATCATAAATATGACCAG |  |  |
|  | 82D26-RNAi-F | CACCCCAGGAATTAAGCAGAAATGATA | Gene knockout | 147 |
|  | 82D26-RNAi-R | AGAGAAATAACAACAGCCCAAAG |  |  |
|  | 82D26qRT-F | AAAGCTACTTGCCTGGGCTT | qRT-PCR | 279 |
|  | 82D26qRT-R | ATGATATCCACCCACGGTGC |  |  |

**Table S6. Enzymatic activity of six soybean CYP82 subfamily proteins toward different flavonoid compounds when feeding in yeast.**

| Type | Substrate | GmCYP82A2 | GmCYP82A3 | GmCYP82A4 | GmCYP82A23 | GmCYP82C20 | GmCYP82D26 |
| --- | --- | --- | --- | --- | --- | --- | --- |
| Flavanone | Dihydromyricetin | - | - | - | - | - | - |
|  | Eriodictyol | - | - | - | - | - | - |
|  | Liquiritigenin | - | ? | - | - | - | - |
|  | Naringenin | - | - | - | - | - | + |
| Flavone | Apigenin | - |  | - | - | - | - |
| Flavonol | Kaempferol | - | - | - | - | - | - |
| Isoflavone | Daidzein | - | - | - | - | - | - |
|  | Genistein | - | - | - | - | - | - |
|  | 2'-Hydroxygenistein | - | - | - | - | - | - |
| Ptercarpan | 3,9-Dihydroxyptercarpan | - | - | - | - | - | - |

**Fig. S1. The diagram of isoflavone biosynthetic pathway in soybean.**

CHS, Chalcone synthase; CHR, Chalcone reductase; CHI, Chalcone isomerase; IFS, Isoflavone synthase; HID, 2-Hydroxyisoflavanone dehydratase; I2’H, Isoflavone 2’-hydroxylase; IFR, Isoflavone reductase; PTS, Pterocarpen synthase; P6αH, Pterocarpan 6α-hydroxylase; G2DT, (-)-Glycinol 2-dimethylallyltransferase; G4DT, (-)-Glycinol 4-dimethylallyltransferase.

Fig. S2. Multiple sequence alignment of soybean CYP82 family genes at amino acid level.

Amino acid sequence alignment of GmCYP82 subfamily were performed by DNAMAN software. Amino acid sequence with similarity 100% are in black background, similarity greater than or equal to 75% are in dark gray background, similarity between 50% and 75% are in light gray background, similarity less than 50% are in white background. The conserved domains are underlined, including proline rich membrane hinge (proline_rich), I-helix (AGxD/ET), K-helix consensus sequence (KETLR), PERF consensus sequence and heme binding domain (FXXGXRXCXG).


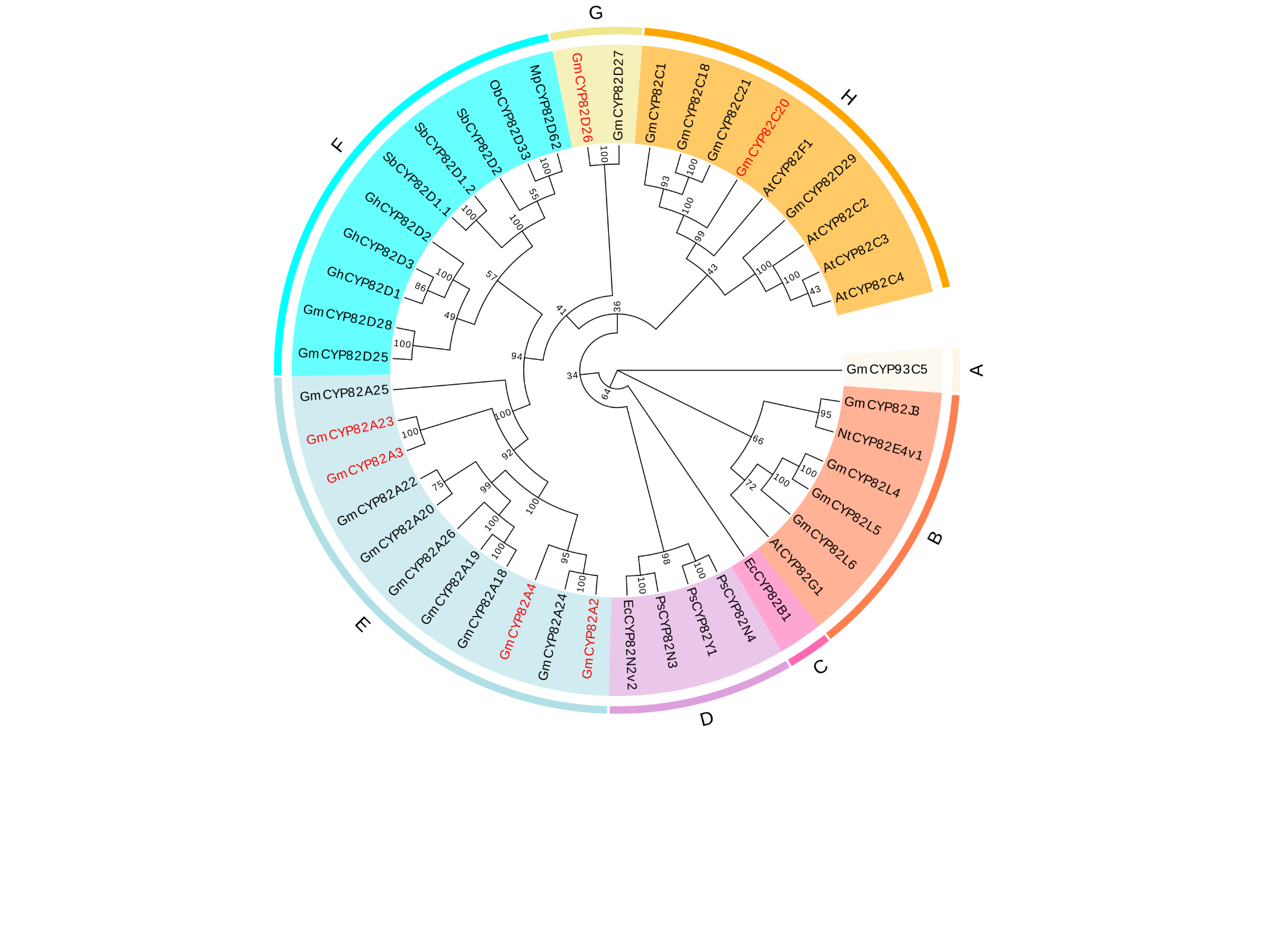


Fig. S3. Phylogenetic tree of CYP82 subfamily members from various plant species.

Maximum-likelihood evolutionary tree was constructed by using Mega-X software with bootstrap value of 1,000. The selected candidate genes in this study were labeled in red. The clades were named by A, B, C, D, E, F, G, H.

**Fig. S4. MS/MS spectrum of the enzymatic product of GmCYP82D26 fed with naringenin.**

(A) MS/MS spectrum of the enzymatic products of GmCYP82D26; (B) MS/MS spectrum of the daidzein standard.

**Fig. S5.** **UPLC profile of the *in vitro* enzymatic products of GmCYP82D26 in yeast microsome with naringenin as substrate.**

**Fig. S6-1. Detection of enzymatic product of the five recombinant GmCYP82 protein with naringenin as substrate by UPLC.**

**Fig. S6-2. Detection of enzymatic product of the six recombinant GmCYP82 protein with eriodictyol as substrate by UPLC.**

**Fig. S6-3. Detection of enzymatic product of the six recombinant GmCYP82 protein with 3,9-dihydroxyptercarpan as substrate by UPLC.**

**Fig. S6-4. Detection of enzymatic product of the six recombinant GmCYP82 protein with genistein as substrate by UPLC.**

**Fig. S6-5. Detection of enzymatic product of the six recombinant GmCYP82 protein with 2'-hydroxygenistein as substrate by UPLC.**

**Fig. S6-6. Detection of enzymatic product of the six recombinant GmCYP82 protein with daidzein as substrate by UPLC.**

**Fig. S6-7. Detection of enzymatic product of the six recombinant GmCYP82 protein with apigenin as substrate by UPLC.**

**Fig. S6-8. Detection of enzymatic product of the six recombinant GmCYP82 protein with liquiritigenin as substrate by UPLC.**

**Fig. S6-9. Detection of enzymatic product of the six recombinant GmCYP82 protein with kaempferol as substrate by UPLC.**

**Fig. S6-10. Detection of enzymatic product of the six recombinant GmCYP82 protein with dihydromyricetin as substrate by UPLC.**


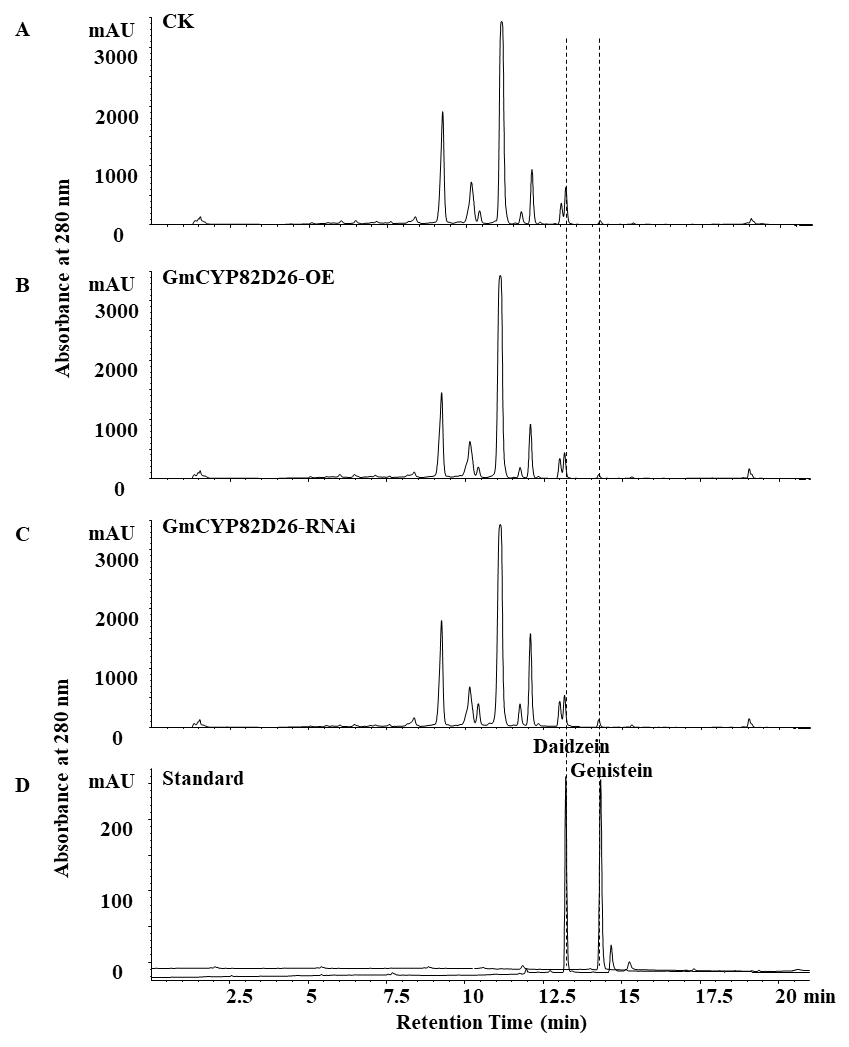


**Fig. S7. UPLC profile of transgenic hairy roots over-expressing and silencing *GmCYP82D26*.**

(A-C) UPLC profiles of flavonoids in transgenic hairy roots detected at wavelength of 280 nm for control (A), *GmCYP82D26* over-expressing lines (B) and RNAi lines (C); (D) UPLC profiles of daidzein and genistein standards.


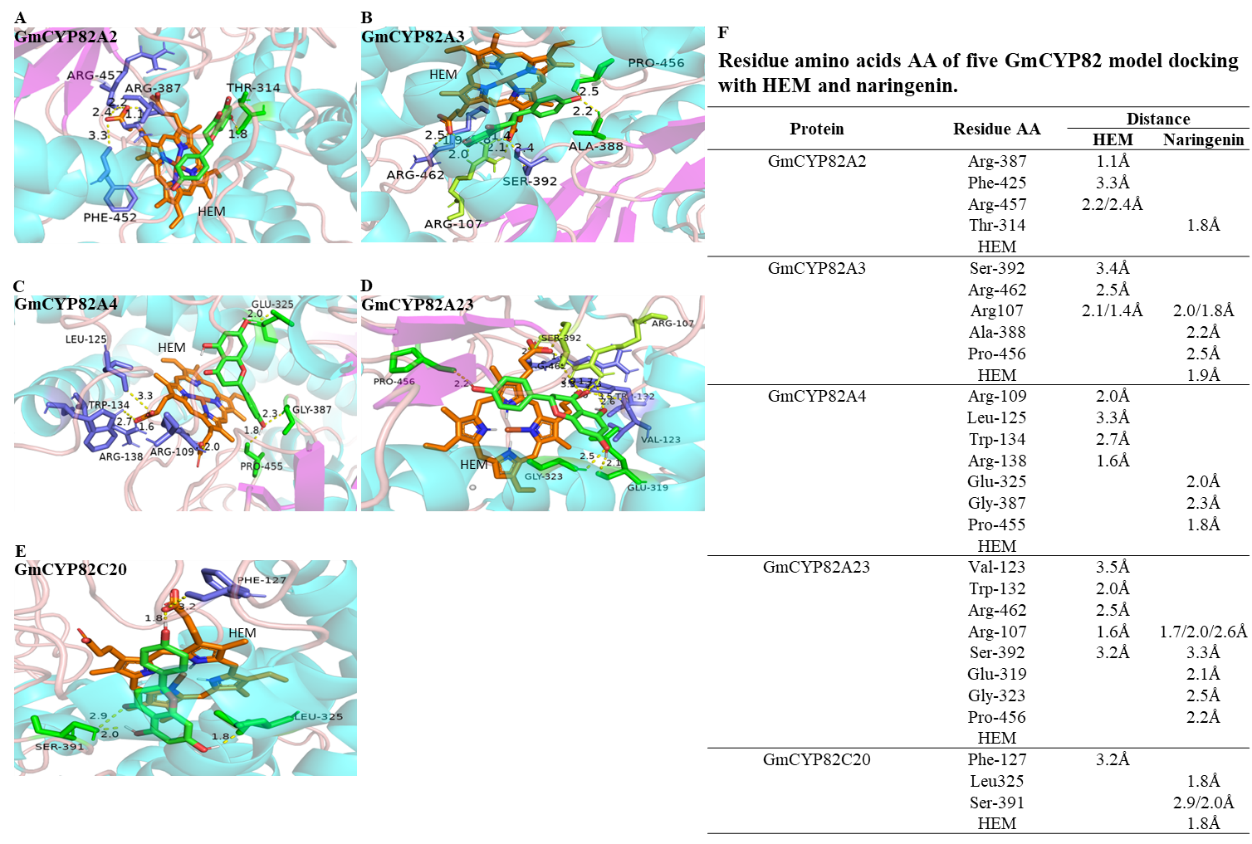


**Fig. S8. Structural modeling of GmCYP82A, GmCYP82A3, GmCYP82A4, GmCYP82A23 and GmCYP82C20 docking with HEM and naringenin.**

The structural models of GmCYP82A2 (A), GmCYP82A3 (B), GmCYP82A4 (C), GmCYP82A23 (D) and GmCYP82C20 (E) docking with naringenin and HEM. The amino acid residues binding with HEM and the substrate of naringenin: HEM was displayed by coral color, naringenin was displayed by green and red color; the amino acid residues binding to HEM are shown in slateblue, the amino acid residues binding to naringenin are shown in limegreen, and the amino acid residues binding to both naringenin and HEM are shown in limon. (F) Residue amino acids AA of five GmCYP82 model docking with HEM and naringenin.
